# The Unexpected Chemistry of Thiacalix[4]arene Monosulfoxide

**DOI:** 10.3390/molecules28093914

**Published:** 2023-05-05

**Authors:** Kamil Mamleev, Václav Eigner, Hana Dvořáková, Pavel Lhoták

**Affiliations:** 1 Department of Organic Chemistry, University of Chemistry and Technology, Prague (UCTP), Technická 5, 166 28 Prague, Czech Republic; 2 Department of Solid State Chemistry, UCTP, Technická 5, 166 28 Prague, Czech Republic; 3 Laboratory of NMR Spectroscopy, UCTP, Technická 5, 166 28 Prague, Czech Republic

**Keywords:** thiacalixarene, spirodienone, oxidation, monosulfoxide, regioisomers, dealkylation, boronate complex, X-ray analysis

## Abstract

Thiacalix[4]arene monosulfoxide **4** possesses a very unusual chemistry, as demonstrated by several unprecedented derivatives in calixarene chemistry. Interestingly, compound **4** cannot be prepared by the dealkylation of its corresponding tetramethoxy derivative using BBr_3_. Instead, the borate complex is formed with a boron bound by the two neighboring phenolic oxygens and a sulfoxide group. A similar type of borate complex with a spirodienone fragment was then isolated as a by-product. The oxidation of monosulfoxide with Chloramine-T did not provide the expected spirodienone moiety, but rather a complex oxathiane-based spiroheterocyclic part containing a chlorine atom. X-ray analyses confirmed the structures of the unusual products and feasible formation mechanisms were proposed. These results provide further evidence of the distinction between thiacalixarene chemistry and the chemistry of classical CH_2_ analogues.

## 1. Introduction

Calixarenes [1,2,3,4,5] represent an integral part of contemporary supramolecular chemistry. Their unique properties predetermine their role as smart building blocks, molecular receptors, or self-assembly systems with a wide range of possible applications. Due to calixarenes’ popularity, there have been rapid developments in the derivatization of these macrocycles, resulting in the very well-established basic chemistry of these systems. Especially in calix[4]arene I, there are a large number of regioselective or stereoselective transformations that enable almost any derivatization of the basic skeleton.

Although thiacalix[4]arene II has been known for more than two decades [6], the chemistry of these sulfur analogues still yields many exciting and surprising discoveries [7,8,9]. The introduction of sulfur bridges into the calixarene skeleton results in a range of new features related to changed complexation properties, different conformational preferences, or an entirely new type of reactivity, which was impossible with classic derivatives or had never been observed.

**Figure 1:**
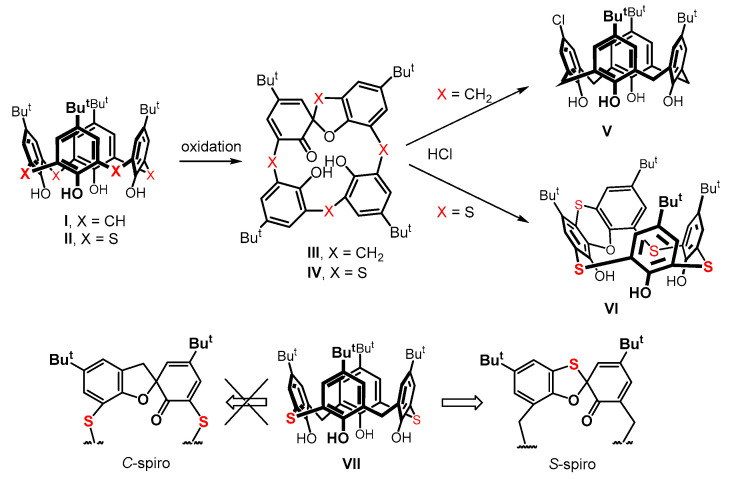
The formation of spirodienone derivatives (**III** and **IV**) of classical calix[4]arene **I** and thiacalix[4]arene **II**, and a different reactivity towards hydrochloric acid; regioselective formation of the ***S*-spiro** isomer from compound **VII**.

An interesting example of dramatically different chemical behavior can be demonstrated in the so-called spirodienone derivatives **III** and **IV**. These compounds are prepared by a careful oxidation of the starting macrocycles (Figure 1). In the classical calixarene series, they represent compelling synthetic intermediates [10,11,12,13,14,15] for the subsequent derivatization of the basic skeleton, enabling the achievement of unusual derivatization patterns. As an example, the reaction of **III** with hydrochloric acid leads to a monochloro derivative [16] of calix[4]arene **V** (Figure 1).

In contrast, the same reaction of the sulfur analog **IV [17]** affords a different product, namely a macrocycle **VI** containing a phenoxathiin group [18,19], as a result of an acid-catalyzed rearrangement. As these compounds represent inherently chiral systems [20,21], the formation of spirodienone derivatives can be useful in the design of new receptors for chiral resolution. In order to understand the rules governing the formation of these spirodienone substances, in our previous work, we studied the oxidation of a system consisting of the alternating CH_2_ and -S- bridges, i.e., on 2,14-dithiacalix[4]arene **VII** [22]. Such a system exhibited a high regioselectivity in the formation of the *S*-spiro isomer, where the bridging sulfur became a part of the spiro moiety (Figure 1).

**Figure 2:**
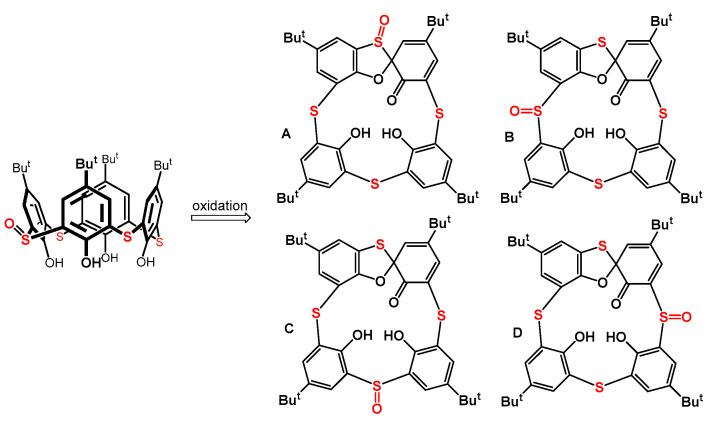
Thiacalix[4]arene monosulfoxide and possible regioisomers of the spirodienone products.

Applying less symmetrical starting compounds for the synthesis of spiro derivatives in this context could form various regio-/stereo- isomers. At the same time, this would also allow us to obtain a greater structural diversity for these substances as chiral building blocks. We used thiacalixarene monosulfoxide (Figure 2) as a starting compound to achieve this goal and investigated its oxidation reactions to determine the formation regioselectivity of the theoretically possible spiro isomers (A–D). This paper summarizes the surprising results that we obtained during this study.

## 2. Results and Discussion

**Scheme 1:**
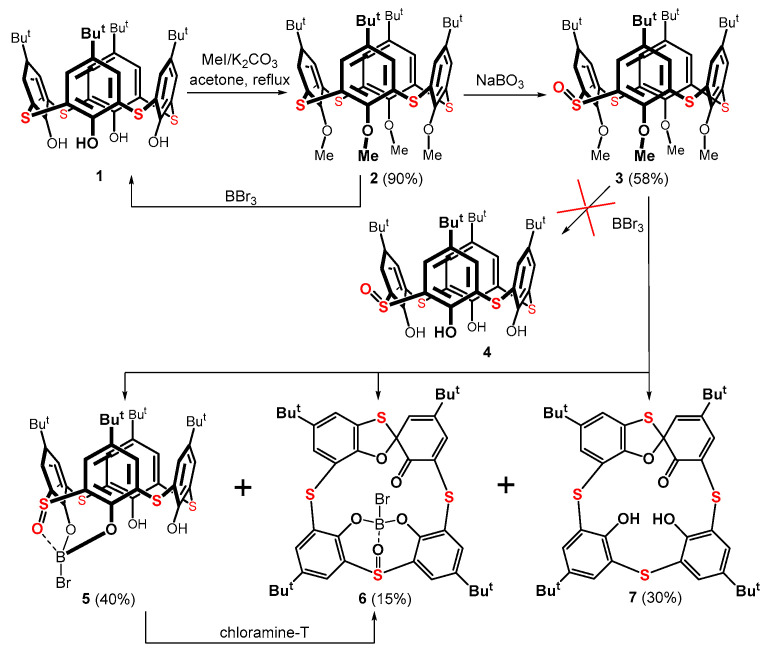
Attempted preparation of thiacalix[4]arene monosulfoxide **4**.

The starting thiacalix[4]arene **1** was first alkylated to the corresponding tetramethyl derivative **2** (Scheme 1) following a published procedure [23]. The oxidation of this compound using sodium perborate produced a mixture of mono- and di-sulfoxides [24]. The required product **3** was obtained using column chromatography on a silica gel in a 58% yield. In the next step, the removal of the methyl ether groups was carried out with boron tribromide, which is frequently used for dealkylation reactions in calixarene chemistry [25,26]. Surprisingly, this reagent did not produce the expected dealkylated thiacalixarene **4**. Instead, a complex reaction mixture was repeatedly formed, where the expected product **4** could not be traced.

A careful separation using column chromatography provided three main products **5**, **6,** and **7** (Scheme 1), along with starting thiacalixarene **1**. The major product **5**, isolated as a white solid in a 40% yield, represents a boronate complex, where a boron atom, bearing one remaining bromine, is bonded to two phenolic oxygens and the sulfoxide group of the thiacalixarene moiety. The HRMS (ESI+) of product **5** showed a peak at *m*/*z* = 847.1402, which is excellent agreement with the calculated value for the [M + Na]^+^ cation of the expected structure (847.1397).

The HRMS peak at *m*/*z* = 845.1254 for compound **6** (isolated as an orange solid in a 15% yield) indicated a similar structure to that of product **5,** but without two hydrogen atoms. Since an orange or orange-red color is a typical feature of thiacalix[4]arene spirodienone derivatives, structure **6** was tentatively assigned as a spiro derivative. The expected mass of [M + Na]^+^ cation for **6** (845.1247) perfectly corresponded with the experimental value.

The last isolated compound **7** (30% yield) of a bright orange color was easily and unambiguously identified by comparing its NMR spectra with those of an authentic sample from our laboratory [18].

**Figure 3:**
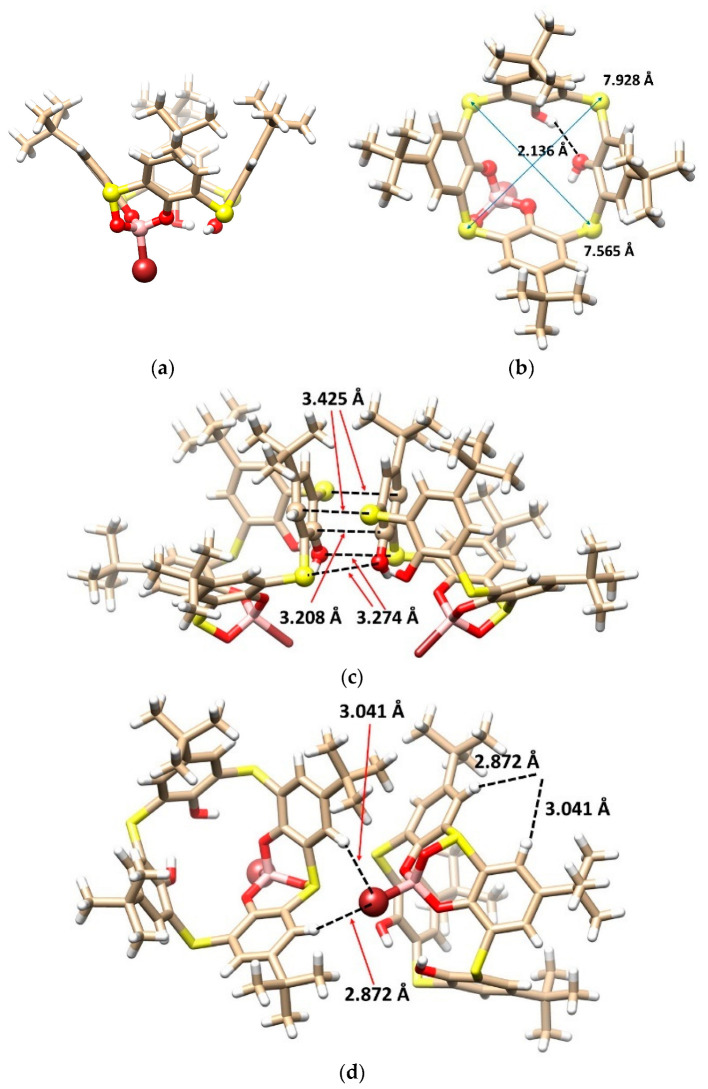
Single crystal X-ray structure of **5**: (**a**) the side view, (**b**) top view showing the O-H···O hydrogen bonding and the main diagonals, (**c**) dimeric motif within the crystal packing based on the O···S, C···S interactions, and π-π interactions, and (**d**) CH···Br interactions in crystal packing (interacting atoms shown as balls for better clarity).

A single crystal X-ray diffraction analysis confirmed the final structure. The boron complex **5** was crystallized in an orthorhombic crystal system, with a space group *Pccn*. As shown in Figure 3a, the molecule adopted the *cone* conformation, which is held by the boronate moiety and one intramolecular hydrogen bond between the phenolic hydroxyls (O···H distance of 2.136 Å). The presence of the boronate function (Ar-O···B distances of 1.442 and 1.446 Å and S=O···B distance of 1.502 Å) made the initial square shape of the cavity somewhat asymmetric, which is shown by the unequal lengths of the two main diagonals (S···S distances between the opposite sulfur atoms of 7.928 and 7.565 Å) (Figure 3b).

An interesting dimeric motif was observed throughout the crystal packing of **5** (Figure 3c), where one can find chalcogen bonding between the oxygen and sulfur atoms (S···O distances of 3.274 Å), together with S···C close contacts between the bridging sulfur and aromatic carbon atoms (S···O distances of 3.425 Å) and π-π interactions between the almost coplanar aromatic rings (C···C distance of 3.208 Å). Another unusual supramolecular bonding motif was represented by the close contacts (Br···H-Ar distances of 2.872 and 3.041 Å) of the bromine atom and two adjacent Ar-H groups (Figure 3d).

**Figure 4:**
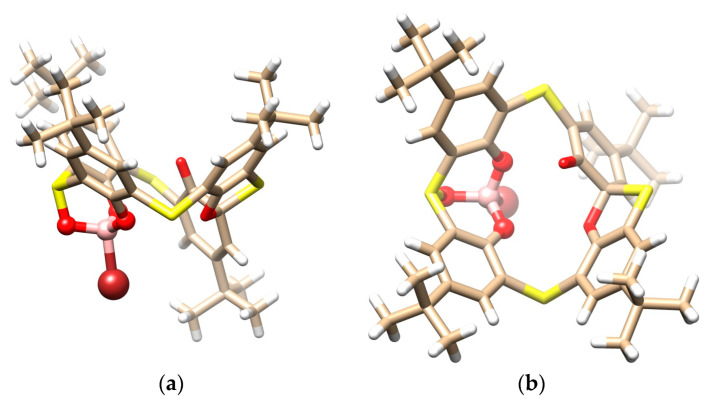

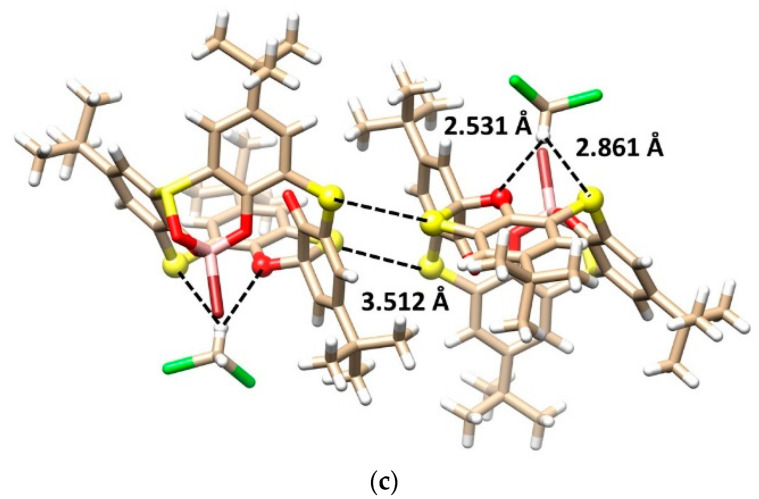
Single crystal X-ray structures of compound **6**: (**a**) the side view and (**b**) the top view, showing the *partial cone* conformation, and (**c**) dimeric motif within the crystal packing based on the S···S interactions between opposite enantiomers + bonding of CH_2_Cl_2_ (interacting atoms shown as balls for better clarity).

Product **6** was crystallized in a triclinic crystal system with a space group *P*-1 and adopted the *partial cone* conformation (Figure 4a,b), with the carbonyl group pointing to the opposite side of the molecule. As a consequence of the spirodienone moiety presence, the compound was chiral. Derivative **6** crystallized as a racemic mixture, where opposite enantiomers are held together via S···S interactions (S···S = 3.512 Å) to form a dimeric motif within the crystal packing (Figure 4c). Moreover, the compound forms a 1:1 solvate with a dichloromethane molecule, which is held outside of the calixarene cavity by the hydrogen bonding interactions between the oxygen atom (CH···O = 2.531 Å) of the spiro group and the neighboring sulfur bridge (CH···S = 2.861 Å) (Figure 4c).

The formation of product **5** was unforeseen. To our knowledge, only one example describes a similar structure in the thiacalixarene series [27]. The rarity of this structural motif is further emphasized by the fact that only one similar arylboronate complex is known in non-calixarene chemistry [28]. Thus, thiacalix[4]arene tetrasulfoxide reacts with Et_3_B in a toluene–DMSO mixture at reflux overnight (or even for several days) to produce the corresponding Et-B(-O-)_3_ complex [27]. It should be noted that the authors used the starting derivative with free OH groups; in our case, this was a methoxy derivative. This means that the boron complex **5** could have formed only after the dealkylation (or during the dealkylation) of the lower rim, and at the same time, it was created under remarkably mild conditions.

We performed some model reactions to determine the formation mechanism of complex **5**. The reaction of **1** with BBr_3_ under exact conditions did not provide any products, while the same reaction of methoxy derivative **2** smoothly led to the dealkylated starting calixarene **1**. In neither case was the formation of any boronate observed. This highlights the crucial role of the sulfoxide moiety in the complex formation. However, the model reaction of non-alkylated monosulfoxide **4** (vide infra) with BBr_3_ yielded only 0.3% of the expected boron complex **5**, suggesting the key influence of methoxy groups during its formation.

The formation of compounds **6** and **7** is even more astonishing. It is evident that, in addition to the formation of the boronate complex, there is also a spirodienone arrangement in **6**. Since the formation of the spiro group is associated with the oxidation of the system, it was not clear what the oxidizing agent was in this case. The reaction was carried out in an inert atmosphere to exclude oxygen. Since neither BBr_3_ nor CH_2_Cl_2_ can be oxidizing agents, the only possible explanation lies in the involvement of a sulfoxide group in starting **3** as an oxidizing agent. Indeed, a considerable amount of the starting sulfoxide was deoxygenated to the sulfide bridge. In addition to compound **7** (30%), the starting thiacalixarene **1** was also isolated (ca 10%) from the reaction mixture. Therefore, the hypothesis of a sulfoxide group being an oxidizing agent is possible.

**Scheme 2:**
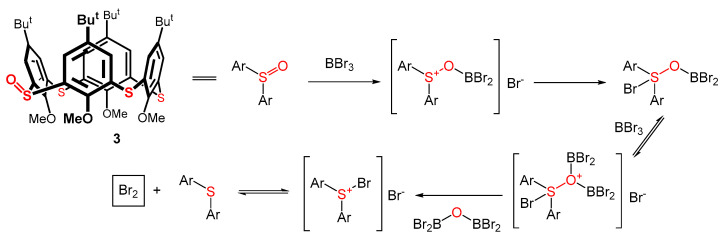
Tentative mechanism of the deoxygenation of thiacalix[4]arene monosulfoxide **3** and the formation of bromine molecule (adapted from Ref. [31]).

The deoxygenation of sulfoxides using boron reagents is known in organic chemistry [29,30]. For example, in 1984, the reaction of dimethylboron bromide or 9-BBN-Br with diphenyl sulfoxide was described [31]. The resulting diphenyl sulfide was isolated with a very high yield, and a similar result (albeit with a slightly lower yield) was also observed for BBr_3_. Interestingly, the authors hypothesized the formation of a bromine Br_2_ during the deoxygenation reaction. The synthesis of calixarene spirodienones using the tribromide anion Br_3_^-^ has already been described [14,32]; hence, the Br_2_ molecule could represent our missing oxidizing agent. The tentative deoxygenation mechanism of monosulfoxide **3** (and the formation of an oxidizing agent Br_2_) is depicted in Scheme 2. This deoxygenation runs as a competing reaction to the dealkylation of the lower rim of the macrocycle.

**Scheme 3:**
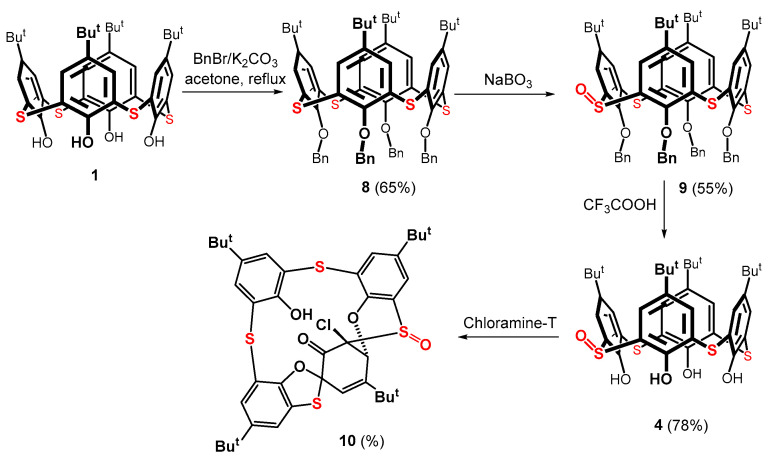
Preparation of thiacalix[4]arene monosulfoxide **4** and its subsequent oxidation.

Because BBr_3_ was unsuitable for the demethylation of thiacalixarene **3**, we tried the other dealkylating agents known from calixarene chemistry. Nevertheless, none of the agents (trimethylsilyl iodide and pyridinium hydrobromide) worked for this intended transformation. Based on these eye-opening outcomes, a different route was chosen to synthesize monosulfoxide **4**. As shown in Scheme 3, the benzylation of compound **1** with benzyl bromide generated the corresponding benzyloxy derivative **8** in a 65% yield [33]. This compound was oxidized by sodium perborate to produce monosulfoxide **9** in a 55% yield after column chromatography on a silica gel [34]. The final debenzylation step was carried out under acidic conditions using TFA. The expected product **4** was obtained in a 78% yield.

**Figure 5:**
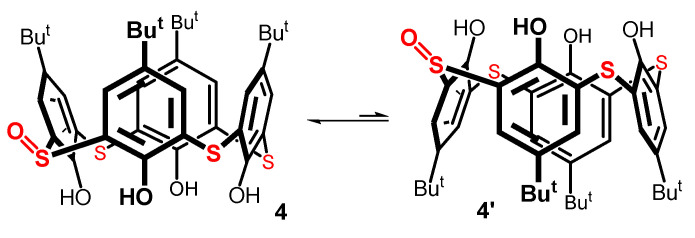

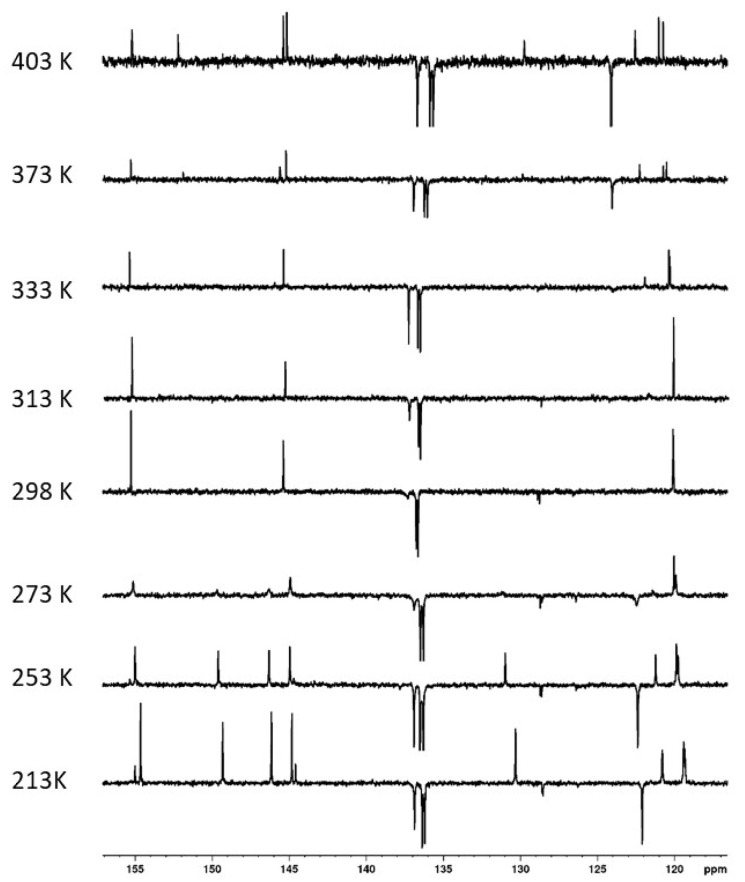
VT NMR experiments showing a dynamic behavior of compound **4**: The aromatic part of ^13^C {^1^H} spectra, 213–298 K in CDCl_3_ and 298–403 K in C_2_D_2_Cl_4_.

While the structure of compound **4** was unequivocally confirmed by HRMS (ESI+) spectra (found *m*/*z* 759.2275 /calc. 759.2277 for [M + Na]^+^ ion), the ^1^H NMR spectra (400 MHz, CDCl_3_) measured at room temperature showed broad peaks, indicating some dynamic phenomena associated with this molecule. Moreover, at room temperature, the ^13^C NMR exhibited six signals in the aromatic part of the spectrum (quaternary + CH aromatic carbons), which is only half the expected number. In order to shed some light on this matter, we studied the dynamic behavior of compound **4** using the variable temperature (VT) NMR technique. As can be seen in Figure 5, the ^13^C NMR spectrum in C_2_D_2_Cl_4_ at 298 K shows three signals for quaternary aromatic carbons and three poorly resolved signals for CH aromatic carbons. As the temperature increased, new peaks gradually appeared. At the highest temperature (403 K), all 12 expected signals in the aromatic region were finally visible (Figure 5, the uppermost spectrum). Fascinatingly, lowering the temperature to 213 K eventually led to the same 12 signals being observed in the aromatic part (three small peaks at 128.5, 144.5, and 155.0 belonging to the starting thiacalixarene **1**, which was formed in the reaction as a difficult-to-separate impurity).

It is known from the literature [35,36] that basic calix[4]arenes maintain a *cone* conformation due to the cyclic array of the intramolecular hydrogen bonds of their phenolic functions. In solution, however, this arrangement rapidly flips to either side of the macrocycle’s main plane, in an equilibrium known as *cone–*(inverted) *cone* interconversion (Figure 5). This phenomenon is also recognized in the chemistry of thiacalix[4]arenes [37]. It is, therefore, highly probable that it occurs in the corresponding monosulfoxide **4**. In the basic thiacalix[4]arene, symmetry makes both the structures identical. In our case, on the contrary, structures **4** and **4’** differ (Figure 5), owing to the presence of the sulfoxide group, which does not change its configuration during the flipping of the macrocycle.

For thiacalixarene derivatives immobilized in the *cone* conformation, it has been reported [38,39] that the sulfoxide group formed by sulfur oxidation always points towards the upper rim of the calixarene (like in **4**). The opposite configuration (like in **4′**) cannot be prepared by direct oxidation, indicating its much lower thermodynamic stability. Applying this knowledge to monosulfoxide **4**, the dynamic behavior can be explained by the rapid equilibrium of the two isomers, **4** and **4’**. The minor isomer **4’** is of such a low abundance that it is not detectable in the NMR spectrum. As expected, the X-ray analysis of **4** (see Figure S33 in SI) revealed a *cone* conformation with the oxygen atom pointing to the upper rim of the macrocycle.

Compound **4** was then oxidized to a monospirodienone derivative using Chloramine-T (*N*-chloro-*p*-toluenesulfonamide sodium salt trihydrate) as an oxidizing agent, following a well-known procedure from calixarene/thiacalixarene chemistry. The reaction of **4** with one equivalent of the agent in a CHCl_3_-MeOH 3:1 *v*/*v* mixture at room temperature led to a highly complex reaction mixture with many close spots on TLC. All of our attempts to influence the reaction outcome by changing the stoichiometry, reaction temperature, and time did not significantly change the crude reaction mixture. Finally, the reaction was carried out under quasi-high-dilution conditions, which provided, after repeated chromatography, a small amount of pure product **10** (10% yield), whose color (yellow-orange) suggested a possible spirodienone arrangement. A HRMS ESI+ analysis showed a peak at *m*/*z* = 791.1737, indicating the chemical formula C_40_H_45_ClO_5_S_4_ + Na^+^. However, this means that the expected spirodienone compound of type **A-D** (C_40_H_46_O_5_S_4_) (Figure 2) did not occur and the resulting structure had an additional chlorine atom instead of hydrogen.

The ^1^H NMR spectrum of **10** (CDCl_3_, 400 MHz) showed eight doublets with *meta* coupling constants (~2 Hz) in the aromatic part of the spectrum, together with a singlet at 6.38 ppm corresponding to the free OH group. By a comparison of this spectrum with that of spiro compound **6**, it is seen that some of the *meta* doublets in compound **10** were at a much stronger field. While the two doublets with the lowest chemical shifts for **6** were at 7.04 and 6.45 ppm, there were four signals for substance **10** with shifts lower than 7 ppm, two of which were at an extremely high field (5.77 and 5.21 ppm), which could indicate a non-aromatic system.

**Figure 6:**
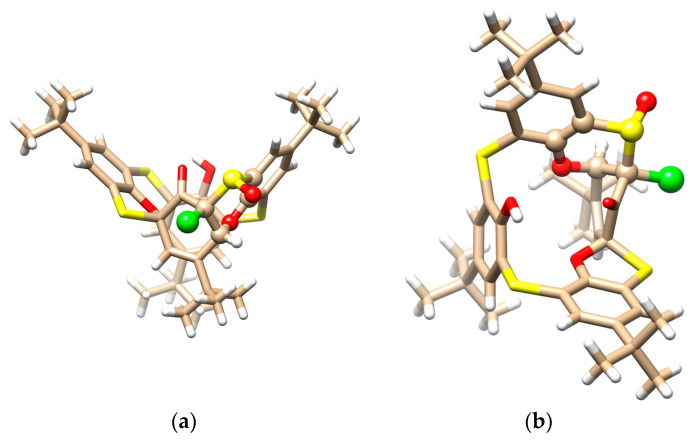

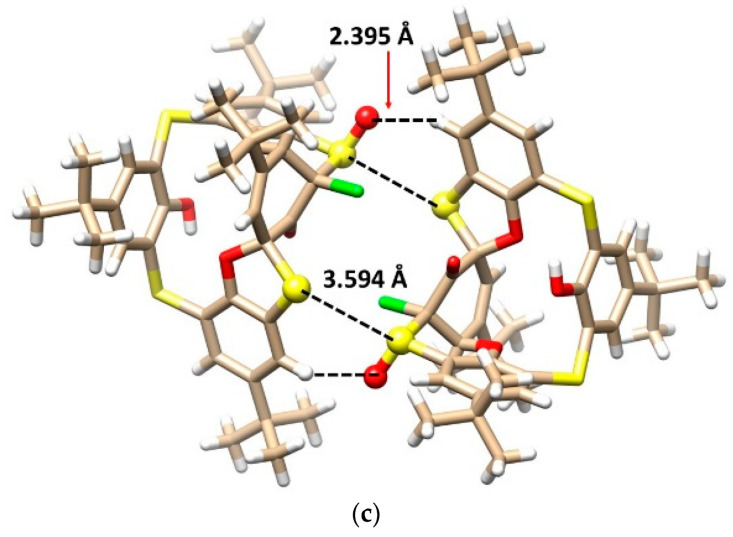
Single crystal X-ray structures of compound **10**: (**a**) side view and (**b**) top view, showing the *1,3-alternate* conformation (oxathiane moiety shown as balls), and (**c**) dimeric motif within the crystal packing (interacting atoms shown as balls for better clarity).

The mystery of the unknown structure was unequivocally solved using a single crystal X-ray diffraction analysis. Compound **10** crystallized in a triclinic crystal system with a space group *P*-1 and formed a CH_2_Cl_2_ solvate with a 2:1 stoichiometry (2 calix:1 DCM). As shown in Figure 6a,b, calixarene adopted the *1,3-alternate* conformation, which is heavily distorted by the spiro arrangement and condensed oxathiane-based heterocyclic moiety containing a chlorine atom. As in the previous cases, a complicated network of supramolecular interactions occurred within the crystal packing. Figure 6c demonstrates the dimeric motif based on the S-S close contacts (S···S = 3.594 Å) and HB interactions of the sulfoxide oxygen with the CH bond of the aromatic unit.

**Scheme 4:**
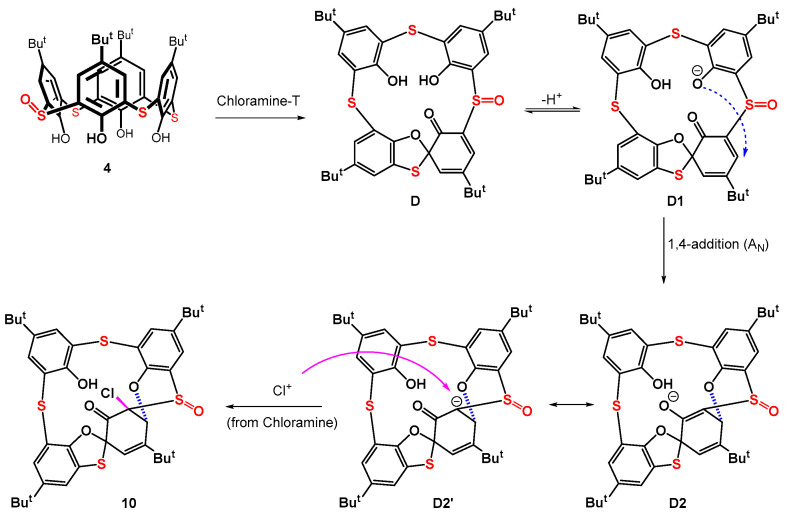
Tentative mechanism of formation of compound **10**.

A possible mechanism for the formation of compound **10** is proposed in Scheme 4. The initially formed spiro compound **D** is deprotonated under the reaction conditions to yield the phenolate analogue **D1**. Supposing compound **D** has a similar spiro structure to compound **6** (see Figure 4 for X-ray), this phenolate anion comes close to the α,β-double bond of the spirodienone moiety and its intramolecular 1,4-conjugate addition can occur. The resulting carbanion/enolate **D2**,**D2’** is then attacked by a Cl^+^ cation (coming from Chloramine-T) from the opposite side of the ring (*anti* addition) to form the final product **10**. Principally similar reactions of α,β-unsaturated ketones with chlorine in methanol [40] and 1,2-benzoquinones with tert-butyl hypochlorite in methanol [41] have been reported in the literature. In both cases, the conjugate addition of the methoxy group and chlorine atom occurs.

## 3. Materials and Methods

### 3.1. General Experimental Procedures

All the commercially obtained chemicals were used as received, without further purification. The solvents were dried and distilled using conventional methods. For the melting point studies, Heiztisch Mikroskop–Polytherm A (Wagner & Munz, München, Germany) was used. The ^1^H, ^13^C{^1^H} NMR spectra were recorded on an Agilent 400-MR DDR2 and JEOL-ECZL400G (^1^H: 400 MHz, ^13^C: 100 MHz). The chemical shifts (δ) are reported in parts per million (ppm) and were referenced to the residual peaks of the solvent or TMS as an internal standard; the coupling constants (J) are expressed in Hz. All the NMR data were processed and displayed using MestReNova software. The IR spectra were measured on an FT–IR spectrometers Nicolet 740 or Bruker IFS66 equipped with a heatable Golden Gate Diamond ATR–Unit (SPECAC) in KBr. A total of 100 scans for one spectrum were co-added at a spectral resolution of 4 cm–1. The electrospray ionization mass spectra (ESI-MS) were recorded using an LTQ Orbitrap Velos-hybrid ion-trap-orbitrap (Thermo Scientific, Waltham, MA, USA). The purity of the substances and courses of the reactions were monitored using thin layer chromatography (TLC), using silica gel 60 F254 on aluminum-backed sheets (Merck) and analyzed at 254 nm. The column chromatography was conducted on silica gel 60 with particle sizes of 0.063–0.200 mm (Merck).

### 3.2. Synthetic Procedures

#### 3.2.1. Tetramethoxy Derivative **2**

The starting thiacalix[4]arene **1** (1.80 g, 2,49 mmol) was dissolved in 75 mL of dry acetone. K_2_CO_3_ (4.14 g, 29.96 mmol) and Iodomethane (3.75 mL, 11.59 mmol) were added and the reaction mixture was stirred and heated at 52 °C overnight. The mixture was cooled to rt and 1M HCl (60 mL) was added, after which the crude product was extracted with chloroform (3 × 40 mL). The organic phase was washed with water and sodium bicarbonate solution, dried over MgSO_4_, and evaporated to dryness. The product was recrystallized from the chloroform/methanol mixture. Tetramethyl derivative **2** was obtained in a 90% (1.74 g) yield as white crystals. All the data are in agreement with the literature [23].

^1^H NMR (CDCl_3_, 400 MHz, 298 K) *δ 7*.43 (s, 8H, Ar-H), 3.44 (s, 12H, -O-CH_3_), and 1.24 (s, 36H, *t*Bu) ppm.

#### 3.2.2. Tetramethoxy Sulfoxide Derivative **3**

Tetramethoxy derivative **2** (1.74 g, 2.24 mmol) was dissolved in 55 mL of CHCl_3_ and 55 mL of AcOH was added. The mixture was heated to 60 °C. Sodium perborate tetrahydrate (1.415 g, 9.0 mmol) was added and the reaction mixture was stirred and heated at 60 °C for 3 h. The reaction mixture was cooled to rt and approx. 150 mL of 1 M HCl was poured into the flask. The crude product was extracted with chloroform (3 × 60 mL), the organic phase was then washed with sodium bicarbonate solution and water, dried over MgSO_4_, and separated using column chromatography on silica gel (eluent = cyclohexane:ethyl acetate = 8:1, *v*/*v*). Tetramethoxy sulfoxide **3** was obtained in a 58% (1.03 g) yield as a white powder. All the data are in agreement with the literature [24].

^1^H NMR (CDCl_3_, 400 MHz, 298 K) *δ* 7.63 (bs, 2H, Ar-H), 7.56 (d, *J* = 2.5 Hz, 2H, Ar-H), 7.43 (bs, 4H, Ar-H), 3.78 (s, 6H, -O-CH_3_), 3.46 (s, 6H, -O-CH_3_), and 1.23 (s, 36H, *t*Bu) ppm.

#### 3.2.3. Attempted Dealkylation of Compound **3**

Tetramethoxy derivative 3 (0.51 g, 0.64 mmol) was dissolved in 25 mL of dry DCM. Boron tribromide (2.7 mL, 1 M in DCM) was added and the reaction mixture was stirred and heated at 33 °C for 2 h under an argon atmosphere. Sodium bicarbonate solution (100 mL) was added, the crude product was extracted with DCM (3 × 50 mL), and the organic phase was then washed with water, dried over MgSO_4_, and separated using column chromatography on silica gel (eluent = cyclohexane:ethyl acetate = 5:1, *v*/*v*). Boron complex 5 was obtained in a 40% (210 mg) yield as a white solid, together with 15% (80 mg) of compound 6 as an orange solid and 30% (140 mg) of compound 7 as an orange solid.

*Data for compound* **5**: m.p. = 303–305 °C. ^1^H NMR (CDCl_3_, 400 MHz, 298 K) *δ* 8.35 (s, 2H, Ar-OH), 8.05 (d, *J* = 2.3 Hz, 2H, Ar-H), 7.62 (d, *J* = 2.5 Hz, 2H, Ar-H), 7.52 (d, *J* = 2.5 Hz, 2H, Ar-H), 7.26 (d, *J* = 2.3 Hz, 2H, Ar-H), 1.29 (s, 18H, *t*Bu), and 1.20 (s 18H, *t*Bu) ppm. ^13^C NMR (CDCl_3_, 100 MHz, 298 K) *δ* 156.65, 151.07, 144.62, 143.66, 141.38, 135.82, 135.40, 127.96, 124.02, 121.62, 118.86, 113.69, 34.67, 34.20, 31.36, and 31.22 ppm. HRMS (ESI^+^) calcd for C_40_H_46_BrO_5_S_4_B 847.1397 [M + Na]^+^, found *m*/*z* 847.1402 [M + Na]^+^.

*Data for compound* **6**: m.p. = 165–168 °C. ^1^H NMR (CDCl_3_, 400 MHz, 298 K) *δ* 7.98 (d, *J* = 2.4 Hz, 1H, Ar-H), 7.59 (d, *J* = 2.3 Hz, 1H, Ar-H), 7.58 (d, *J* = 2.3 Hz, 1H, Ar-H), 7.32 (d, *J* = 2.0 Hz, 1H, Ar-H), 7.18 (d, *J* = 2.4 Hz, 1H, Ar-H), 7.05 (d, *J* = 2.3 Hz, 1H, Ar-H), 7.04 (d, *J* = 2.0 Hz, 1H, Ar-H), 6.54 (d, *J* = 2.2 Hz, 1H, Ar-H), 1.28 (s, 9H, *t*Bu), 1.26 (s 9H, *t*Bu), 1.25 (s, 9H, *t*Bu), and 1.22 (s 9H, *t*Bu) ppm. ^13^C NMR (CDCl_3_, 100 MHz, 298 K) *δ* 189.12, 156.68, 151.51, 147.98, 145.97, 144.77, 143.80, 142.92, 140.64, 132.56, 130.63, 129.27, 128.35, 126.81, 126.23, 125.89, 123.82, 120.87, 120.20, 115.10, 114.64, 113.83, 91.24, 34.85, 34.62, 34.56, 34.52, 31.51, 31.22, 31.18, 28.58, and 27.01 ppm. HRMS (ESI^+^) calcd for C_40_H_44_BrO_5_S_4_B 845.1247 [M + Na]^+^, found *m*/*z* 845.1254 [M + Na]^+^.

*Data for compound* **7**: ^1^H NMR (CDCl_3_, 400 MHz, 298 K) *δ* 8.27 (s, 1H, Ar-OH), 7.81 (s, 1H, Ar-H), 7.64 (d, *J* = 2.5 Hz, 1H, Ar-H), 7.55 (d, *J* = 2.5 Hz, 1H, Ar-H), 7.54 (d, *J* = 2.3 Hz, 1H, Ar-H), 7.45 (d, *J* = 2.5 Hz, 1H, Ar-H), 7.34 (d, *J* = 2.0 Hz, 1H, Ar-H), 7.28 (d, *J* = 2.3 Hz, 1H, Ar-H), 7.04 (d, *J* = 2.1 Hz, 1H, Ar-H), 6.45 (d, *J* = 2.2 Hz, 1H, Ar-H), 1.26 (s, 9H, *t*Bu), 1.24 (s, 9H, *t*Bu), 1.23 (s, 9H, *t*Bu), and 1.22 (s, 9H, *t*Bu) ppm. All the data are in agreement with the literature [24].

#### 3.2.4. Boron Complex **5** (from **4**)

Dealkylated thiacalixarene **4** (0.23 g, 0.31 mmol) was dissolved in 25 mL of dry DCM. Boron tribromide (1.3 mL, 1 M in DCM) was added and the reaction mixture was stirred and heated at 33 °C for 2 days under an argon atmosphere. Sodium bicarbonate solution (75 mL) was added and the crude product was extracted with dichloromethane (3 × 30 mL); the organic phase was then washed with water, dried over MgSO_4_, and separated using column chromatography on silica gel (eluent = cyclohexane: ethyl acetate = 5:1, *v*/*v*). Boron complex **5** was obtained in a 0.3% (7 mg) yield as a white solid. The data are identical with product **5** from the previous reaction.

#### 3.2.5. Reaction of Thiacalixarene **2** with BBr_3_

Thiacalixarene **2** (0.30 g, 0.39 mmol) was dissolved in a mixture of 25 mL of dry dichloromethane and 10 mL of chloroform. Boron tribromide (1.8 mL, 1 M in DCM) was added and the reaction mixture was stirred and heated at 33 °C for 1 day under an argon atmosphere. Sodium bicarbonate solution (75 mL) was added and the crude product was extracted with chloroform (3 × 30 mL); the organic phase was then washed with water, dried over MgSO_4_, and evaporated to dryness. Pure thiacalixarene **1** was obtained in a 99% (0.275 g) yield as a white solid.

^1^H NMR (CDCl_3_, 400 MHz, 298 K) *δ* 9.60 (s, 4H, Ar-OH), 7.64 (s, 8H, Ar-H), and 1.22 (s, 36H, *t*Bu) ppm. The data are in agreement with the literature [6].

#### 3.2.6. Compound **6** (from **5**)

Boron complex **5** (0.11 g, 0.15 mmol) was dissolved in 10 mL of CHCl_3_ and 3 mL of methanol was added. Chloramin T trihydrate (0.04 g, 0.15 mmol) was added and the reaction mixture was stirred at rt. for 1 h. The solvent was evaporated. The residue was dissolved in CH_2_Cl_2_. The organic layer was washed with water and sodium chloride solution, dried over MgSO_4_, and separated using column chromatography on silica gel (eluent = cyclohexane:DCM = 1:3, *v*/*v*). Compound **6** was obtained in a 6% (6.5 mg) yield as an orange solid. The data are identical with product **6** from the previous reaction (Section 3.2.3).

#### 3.2.7. Compound **8**

The starting thiacalix[4]arene **1** (1 g, 1,39 mmol) was dissolved in 14 mL of dry THF. NaH (0.6 g, 60% in oil) and 1.5 mL of DMF were added and the reaction mixture was stirred for 30 min at rt. Benzyl bromide (2.73 mL, 23 mmol) was added and the reaction mixture was stirred for 2 h under reflux. Methanol was added and the solvent was evaporated. The residue was dissolved in CHCl_3_. The organic layer was washed with water, dried over MgSO_4_, and separated using column chromatography on silica gel (eluent = cyclohexane:DCM = 5:1, *v*/*v*). Compound **8** was obtained in a 65% (0.98 g) yield as a white powder.

^1^H NMR (CDCl_3_, 400 MHz, 298 K) *δ* 7.46–7.49 (m, 8H, Ar-H), 7.22 (s, 8H, Ar-H), 7.14–7.20 (m, 12H, Ar-H), 5.24 (s, 8H, -O-CH_2_-Ph), and 1.06 (s, 36H, *t*Bu), ppm. The data are in agreement with the literature [33].

#### 3.2.8. Compound **9**

Compound **8** (0.98 g, 0.9 mmol) was dissolved in 40 mL of CHCl_3_ and 60 mL of AcOH was added. The mixture was heated to 50 °C. Sodium perborate tetrahydrate (0.1 g, 1 mmol) was added and the reaction mixture was stirred and heated at 50 °C overnight. The crude product was extracted with chloroform (3 × 50 mL) and the organic phase was then washed with brine solution and water, dried over MgSO_4_, and separated using column chromatography on silica gel (eluent = cyclohexane: ethyl acetate = 4:1, *v*/*v*). Compound **9** was obtained in a 55% (0.54 g) yield as a white powder.

^1^H NMR (CDCl_3_, 500 MHz, 298 K) *δ* 7.52–7.57 (m, 4H, Ar-H), 7.41 (d, *J* = 2.5 Hz, 2H, Ar-H), 7.40 (d, *J* = 2.4 Hz, 2H, Ar-H), 7.29–7.34 (m, 4H, Ar-H), 7.17–7.25 (m, 16H, Ar-H), 5.54 (d, *J* = 11.6 Hz, 2H, -O-CH_2_(A)-Ph), 5.27 (d, *J* = 11.0 Hz, 2H, -O-CH_2_(B)-Ph), 5.20 (d, *J* = 11.0 Hz, 2H, -O-CH_2_(B’)-Ph), 5.11 (d, *J* = 11.6 Hz, 2H, -O-CH_2_(A’)-Ph), 1.11 (s, 18H, *t*Bu), and 1.04 (s, 18H, *t*Bu) ppm. The data are in agreement with the literature [34].

#### 3.2.9. Thiacalixarene **Monosulfoxide 4**

Compound **9** (0.54 g, 0.5 mmol) was dissolved in 8 mL of benzene and 14 mL of CF_3_COOH was added. The reaction mixture was stirred and heated at 60 °C for 4 days. Water (20 mL) was added and the crude product was extracted with CHCl_3_ (3 × 40 mL). The organic layer was washed with water, sodium bicarbonate solution, and sodium chloride solution. The organic layer was dried over MgSO_4_. The product was recrystallized in the solution of chloroform/methanol. Dealkylated thiacalixarene **4** was obtained in a 78% (0.29 g) yield as white crystals, m.p. = 286–289 °C.

^1^H NMR (CDCl_3_, 500 MHz, 403 K) *δ* 9.19 (br, 4H, OH), 7.55–7.75 (m, 1H, 8H, Ar-H), 1.28 (s, 18H, *t*Bu), and 1.27 (s, 18H, *t*Bu). ^13^C-NMR (CDCl_3_, 125.7 MHz, 403 K) *δ* 155.91, 152.21, 145.39, 145.16, 136.70 135.88, 135.67, 129.76, 124.12, 122.56, 121.03, 120.74, 34.54, 34.20, 31.23, and 31.16. HRMS (ESI^+^) calcd for C_40_H_48_O_5_S_4_ 759.2277 [M + Na]^+^, found *m*/*z* 759.2275 [M + Na]^+^.

#### 3.2.10. Thiacalixarene **10**

Dealkylated thiacalixarene **4** (0.3 g, 0.4 mmol) was dissolved in 30 mL of CHCl_3_ and 10 mL of methanol (solution 1). In another flask, a solution of chloramine T trihydrate (114 mg, 0.4 mmol) in 30 mL of CHCl_3_ and 10 mL of methanol was prepared (solution 2). Using a dual syringe pump, these two solutions were added dropwise into a mixture of 120 mL of CHCl_3_ and 40 mL of methanol at rt over three hours (pseudo-high dilution conditions). After 1 h of stirring at rt, the solvent was evaporated and the residue was dissolved in CH_2_Cl_2_. The organic layer was washed with water and sodium chloride solution, dried over MgSO_4_, and separated using column chromatography on silica gel (eluent = cyclohexane: ethyl acetate = 10:1, *v*/*v*). Compound **10** was obtained in a 10% (31.6 mg) yield as a yellow-orange powder, m.p. 221–223 °C.

^1^H NMR (CDCl_3_, 400 MHz, 298 K) *δ* 7.87 (d, *J* = 2.4 Hz, 1H, Ar-H), 7.59 (d, *J* = 2.3 Hz, 1H, Ar-H), 7.37 (d, *J* = 2.4 Hz, 1H, Ar-H), 7.35 (d, *J* = 2.4 Hz, 1H, Ar-H), 7.00 (d, *J* = 1.9 Hz, 1H, Ar-H), 6.92 (d, *J* = 1.9 Hz, 1H, Ar-H), 6.38 (s, 1H, Ar-OH), 5.77 (d, *J* = 2.0 Hz, 1H, Ar-H), 5.21 (d, *J* = 1.9 Hz, 1H, Ar-H), 1.35 (s, 9H, *t*Bu), 1.28 (s, 9H, *t*Bu), 1.26 (s, 9H, *t*Bu), and 1.17 (s, 9H, *t*Bu) ppm. ^13^C-NMR (CDCl_3_, 100 MHz, 298 K) *δ* 193.09, 154.54, 150.0, 149.31, 147.58, 146.83, 146.38, 142.69, 135.46, 132.77, 128.98, 128.14, 125.51, 124.31, 123.69, 123.04, 122.93, 118.82, 117.58, 117.17, 116.77, 89.89, 75.99, 68.73, 35.61, 34.74, 34.67, 34.29, 31.61, 31.40, 31.22, 29.74, and 28.08. HRMS (ESI^+^) calcd for C_40_H_45_ClO_5_S_4_ 791.1731 [M + Na]^+^, found *m*/*z* 791.1735 [M + Na]^+^.

### 3.3. X-ray Measurements

#### 3.3.1. Crystallographic Data for **4**

M = 737.08 g·mol^−1^, with a tetragonal system, a space group *P*4/*n*, *a* = 15.9156 (3) Å, *c* = 8.2651(2) Å, Z = 2, V = 2093.60 (9) Å^3^, *D_c_* = 1.169 g·cm^−3^, *μ*(Cu-Kα) = 2.39 mm^−1^, and crystal dimensions of 0.41 × 0.30 × 0.27 mm. The data were collected at 180 (2) K on a Bruker D8 Venture Photon CMOS diffractometer with Incoatec microfocus sealed tube Cu-Kα radiation. The data were integrated, scaled, and corrected for absorption using Apex4 [42]. The structure was solved using SHELXT [43] and anisotropically refined using full matrix least squares on F squared using the CRYSTALS [44], with final values of R = 0.045 and wR = 0.124 using 2079 independent reflections (*θ_max_* = 72.3°), 203 parameters, and 90 restrains. The hydrogen atoms bonded to carbon atoms were placed in calculated positions refined with riding constrains, while the hydrogen atoms bonded to oxygen were refined using soft restraints. The disordered functional groups’ positions were found in difference electron density maps and refined with restrained geometry. MCE [45] was used for a visualization of these electron density maps. The occupancy of the disordered functional groups was constrained to full. A suitable model for the disordered mixed solvent could not be found; therefore, its contribution to the structure factors was evaluated using PLATON squeeze [46]. The structure was deposited into the Cambridge Structural Database under the number CCDC 2251514. The full numbering scheme can be found in the Supporting Information Figure S1.

#### 3.3.2. Crystallographic Data for **5**

M = 825,78 g·mol^−1^, with an orthorhombic system, a space group *Pccn*, *a* = 13.29515 (11) Å, *b* = 21.7069 (2) Å, *c* = 31.7636 (3) Å, Z = 8, V = 9166.88 (14) Å^3^, *D_c_* = 1.197 g·cm^−3^, *μ*(Cu-Kα) = 3.22 mm^−1^, and crystal dimensions of 0.62 × 0.07 × 0.07 mm. The data were collected at 95 (2) K on a Rigaku OD Supernova Atlas S2 CCD detector equipped with a mirror collimated microfocused sealed X-ray tube. The data were integrated, scaled, and corrected for absorption using CrysAlis PRO [47]. The structure was solved using charge-flipping methods [48] and anisotropically refined using full matrix least squares on F squared using the CRYSTALS [44], with final values of R = 0.040 and wR = 0.106 using 9210 independent reflections (*θ_max_* = 74.2°), 506 parameters, and 59 restrains. The hydrogen atoms bonded to carbon atoms were placed in calculated positions refined with riding constrains, while the hydrogen atoms bonded to oxygen were refined using soft restraints. The disordered functional groups’ positions were found in difference electron density maps and refined with restrained geometry. MCE [45] was used for a visualization of these electron density maps. The occupancy of the disordered functional groups was constrained to full. A suitable model for the disordered solvent could not be found; therefore, its contribution to the structure factors was evaluated using PLATON squeeze [46]. The structure was deposited into the Cambridge Structural Database under the number CCDC 2251515. The full numbering scheme can be found in the Supporting Information Figure S2.

#### 3.3.3. Crystallographic Data for **6**

M = 908.72 g·mol^−1^, with a triclinic system, a space group *P-1*, *a* = 10.4292 (9) Å, *b* = 13.5249 (12) Å, *c* = 17.3563 (14) Å, *α* = 102.140 (4)°, *β* = 101.407 (4)°, *γ* = 112.044 (4)° Z = 2, V = 2112.5 (3) Å^3^, *D_c_* = 1.429 g·cm^−3^, *μ*(Cu-Kα) = 4.68 mm^−1^, and crystal dimensions of 0.29 × 0.14× 0.08 mm. The data were collected at 180 (2) K on a Bruker D8 Venture Photon CMOS diffractometer with Incoatec microfocus sealed tube Cu-Kα radiation. The data were integrated, scaled, and corrected for absorption using Apex4 [42]. The structure was solved using charge-flipping methods [48] and anisotropically refined using full matrix least squares on F squared using the CRYSTALS [44], with final values of R = 0.089 and wR = 0.254 using 7760 independent reflections (*θ_max_* = 68.9°), 576 parameters, and 81 restrains. The hydrogen atoms bonded to carbon atoms were placed in calculated positions refined with riding constrains, while the hydrogen atoms bonded to oxygen were refined using soft restraints. The disordered functional groups’ positions were found in difference electron density maps and refined with restrained geometry. MCE [45] was used for a visualization of these electron density maps. The occupancy of the disordered functional groups was constrained to full. The structure was deposited into the Cambridge Structural Database under the number CCDC 2251516. The full numbering scheme can be found in the Supporting Information Figure S3.

#### 3.3.4. Crystallographic Data for **10**

M = 1623.99 g·mol^−1^, with a triclinic system, a space group *P-1*, *a* = 9.7262 (4) Å, *b* = 13.3623 (6) Å, *c* = 17.6266 (7) Å, *α* = 73.3923 (15)°, *β* = 85.3415 (14)°, *γ* = 69.9635 (14)° Z = 1, V = 2062.04 (15) Å^3^, *D_c_* = 1.308 g·cm^−3^, *μ*(Mo-Kα) = 0.40 mm^−1^, and crystal dimensions of 0.20 × 0.19 × 0.10 mm. The data were collected at 180 (2) K on a Bruker D8 Venture Photon CMOS diffractometer with Incoatec microfocus sealed tube Mo-Kα radiation. The data were integrated, scaled, and corrected for absorption using Apex4 [42]. The structure was solved using charge-flipping methods [48] and anisotropically refined using full matrix least squares on F squared using the CRYSTALS [44], with final values of R = 0.039 and wR = 0.115 using 8352 independent reflections (*θ_max_* = 26.4°), 606 parameters, and 146 restrains. The hydrogen atoms bonded to carbon atoms were placed in calculated positions refined with riding constrains, while the hydrogen atoms bonded to oxygen were refined using soft restraints. The disordered functional groups and solvent positions were found in difference electron density maps and refined with restrained geometry. MCE [45] was used for a visualization of these electron density maps. The occupancy of the disordered functional groups was constrained to full. The structure was deposited into the Cambridge Structural Database under the number CCDC 2251517. The full numbering scheme can be found in the Supporting Information Figure S4.

## 4. Conclusions

Thiacalix[4]arene monosulfoxide **4** possesses a very unusual chemistry, as demonstrated by several unprecedented derivatives in calixarene chemistry. Interestingly, compound **4** cannot be prepared with the dealkylation of its corresponding tetramethoxy derivative using BBr_3_. Instead, the borate complex is formed with boron bound by the two neighboring phenolic oxygens and a sulfoxide group. A similar type of borate complex with a spirodienone fragment was then isolated as a by-product. The oxidation of monosulfoxide with Chloramine-T did not provide the expected spirodienone moiety, but rather a complex oxathiane-based spiroheterocyclic moiety containing a chlorine atom. X-ray analyses demonstrated the structures of these unique products and the possible mechanisms for forming these compounds were proposed. These results represent further evidence showing how much the chemistry of thiacalixarenes can differ from that of classical CH_2_ analogues.

## Data Availability

All experimental data are provided in the Supplementary information.

## Supplementary Materials

The following supporting information can be downloaded at: https://www.mdpi.com/article/10.3390/molecules28093914/s1, Spectral characterization of all new compounds (^1^H NMR, ^13^C NMR, HRMS, IR), VT NMR experiments and the X-ray structures.

## Sample Availability

Samples of the compounds are not available from the authors.
