# Supplementary material for: The Unexpected Chemistry of Thiacalix[4]arene Monosulfoxide
Source: Molecules. 2023 May 5;28(9):3914. doi: 10.3390/molecules28093914 (PMC10179807; doi:10.3390/molecules28093914)
Supplement: Supplementary file 1 [file molecules-28-03914-s001.zip › molecules-2341516-supplementary.pdf]

Supporting Information

Unexpected Chemistry of Thiacalix[4]arene Monosulfoxide

Kamil Mamleev[1], Václav Eigner[2], Hana Dvořáková[3] and Pavel Lhoták[1,*]

Table of Contents

1. Spectral characterization of compounds 2

2. The Variable Temperature (VT) NMR experiments 11

3. Crystallographic data 19

1. Spectral characterization of compounds

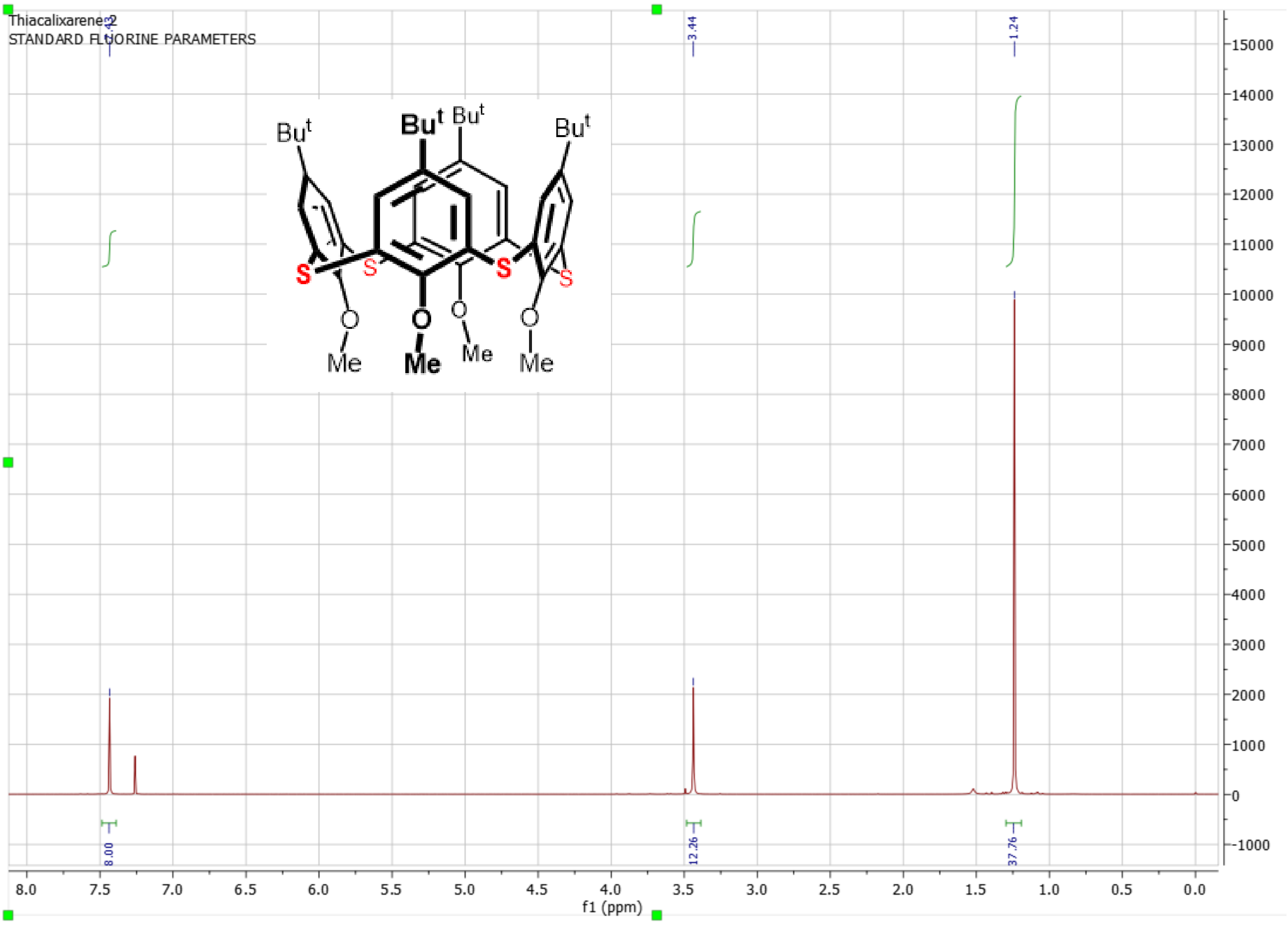

Figure S1: ^{1}H NMR of compound tetramethoxy derivative **2** ($CDCl_3$, 400 MHz).

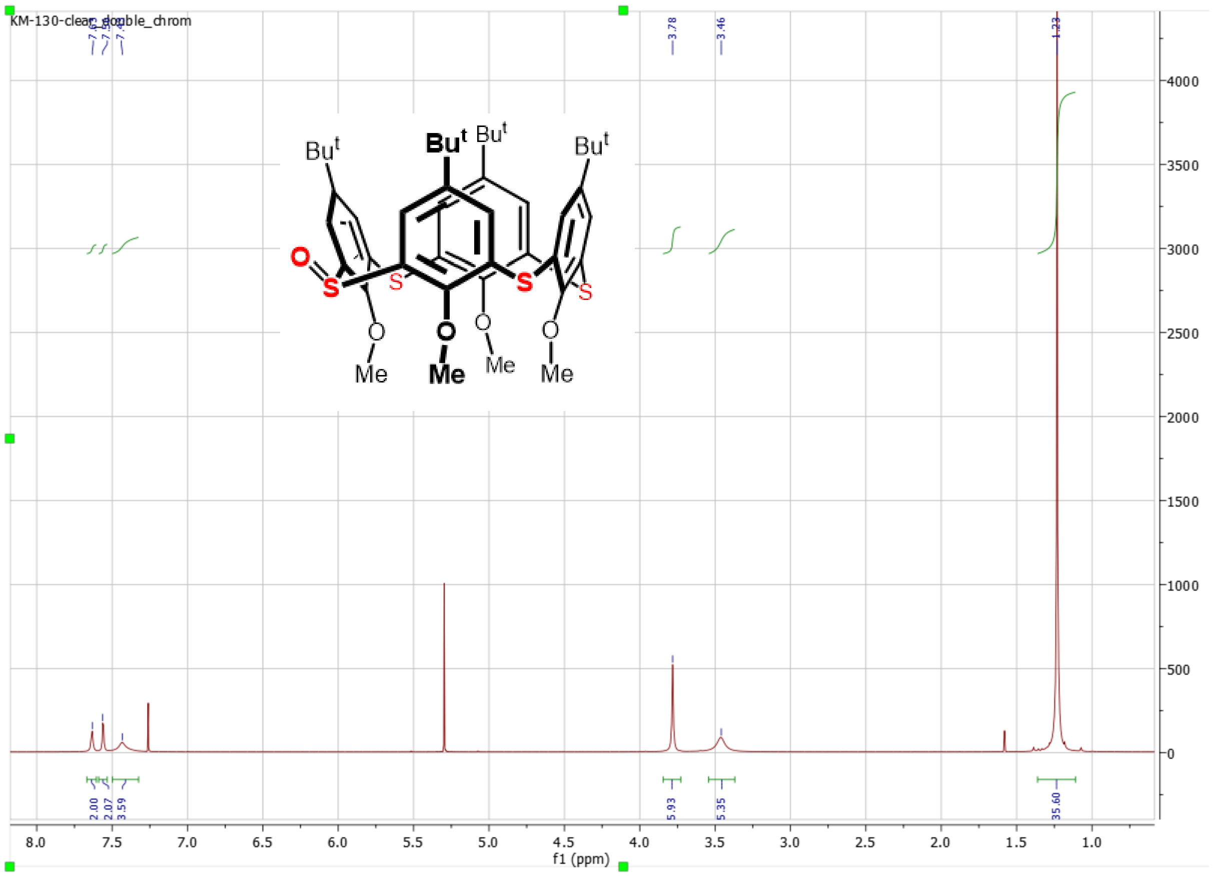

Figure S2: ^{1}H NMR of compound **3** ($CDCl_3$, 400 MHz).

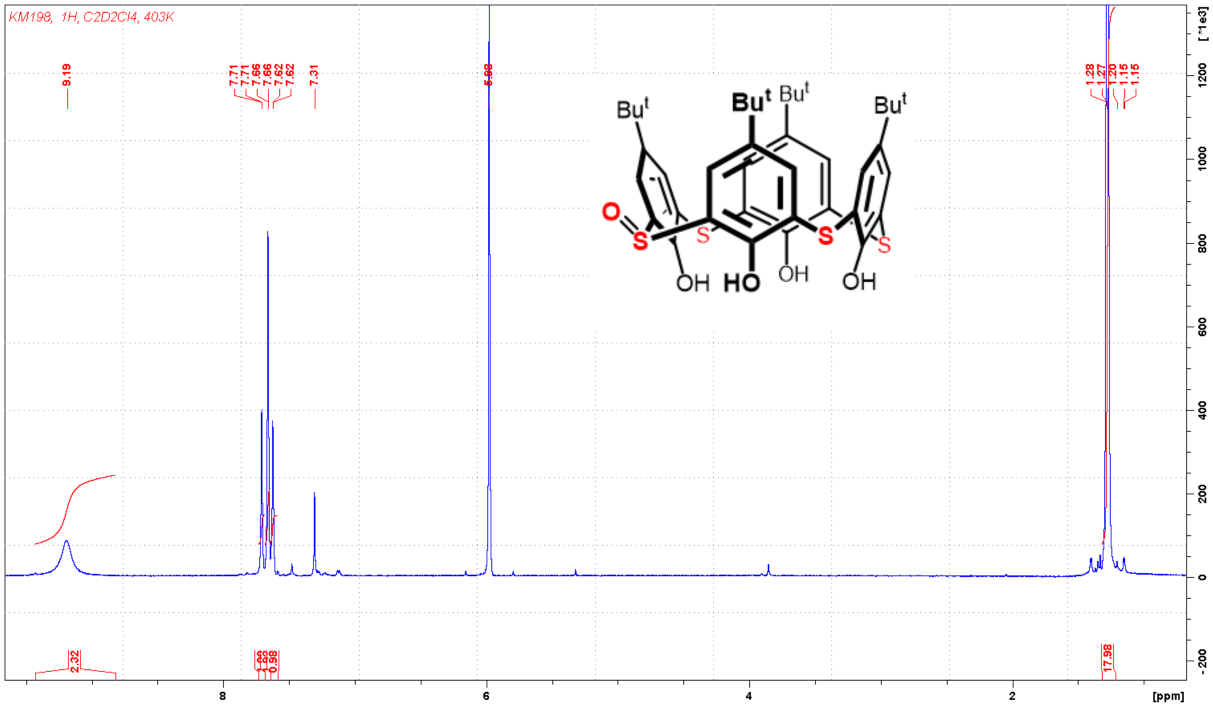

Figure S3: ^{1}H NMR of compound **4** ($C_2D_2Cl_4$, 500 MHz, 403 °K).

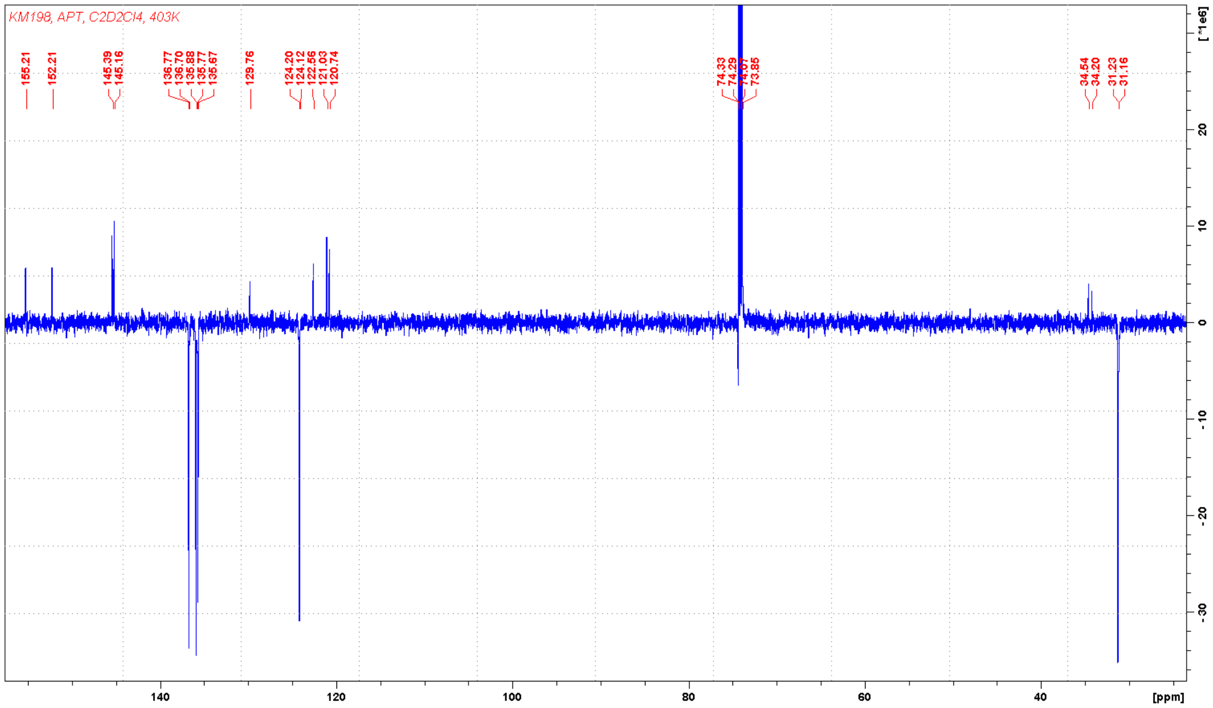

Figure S4: ^{13}C(APT) NMR of compound **4** ($C_2D_2Cl_4$, 100 MHz, 403 °K)

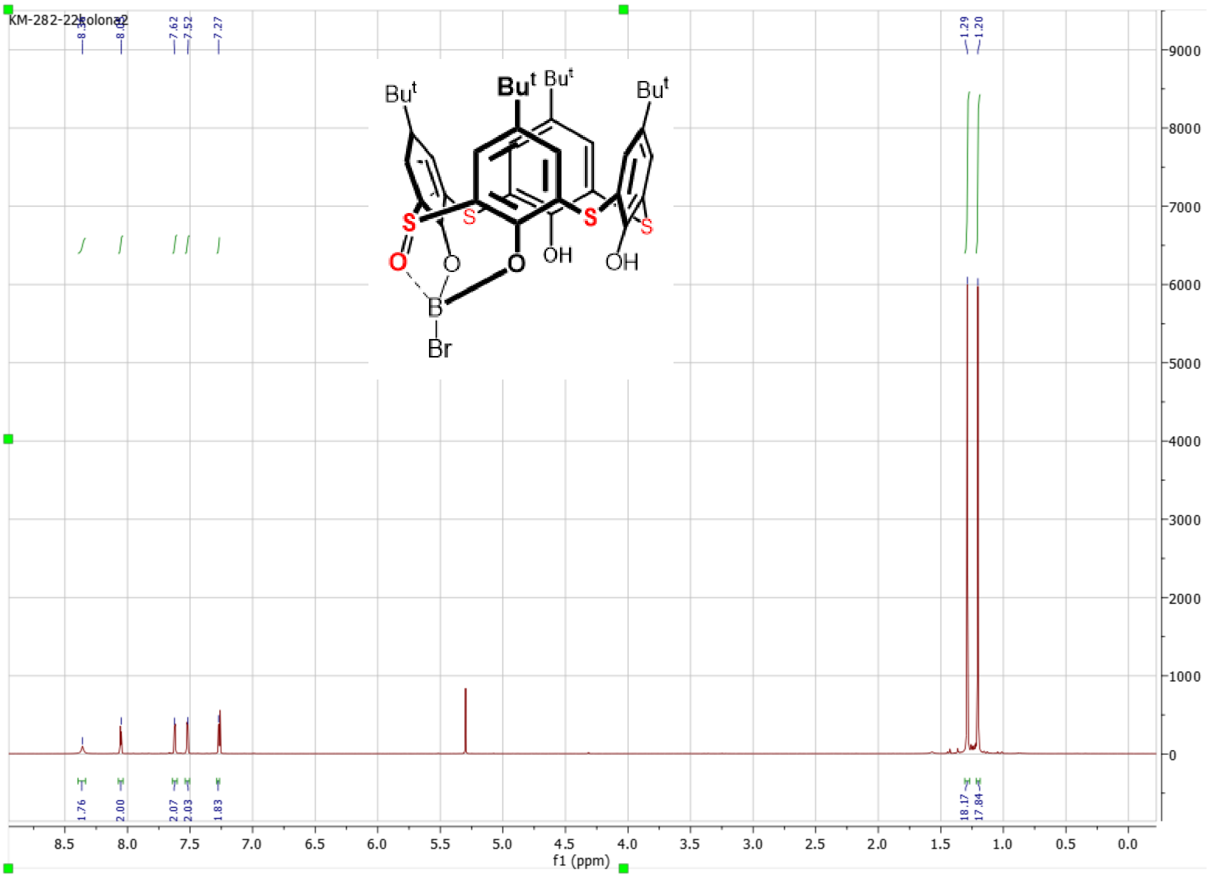

Figure S5: ^{1}H NMR of compound **5** ($CDCl_3$, 400 MHz).

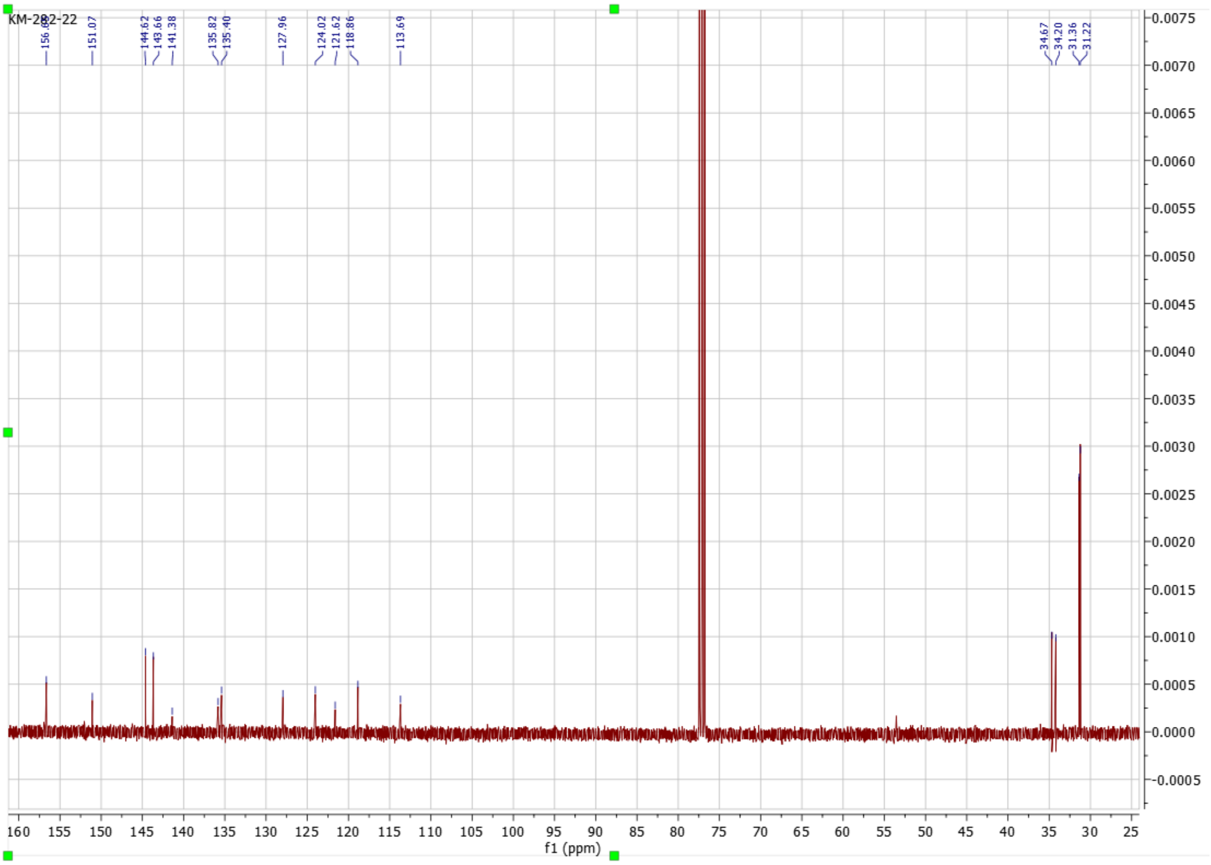

Figure S6: ^{13}C NMR of compound **5** ($CDCl_3$, 100 MHz)

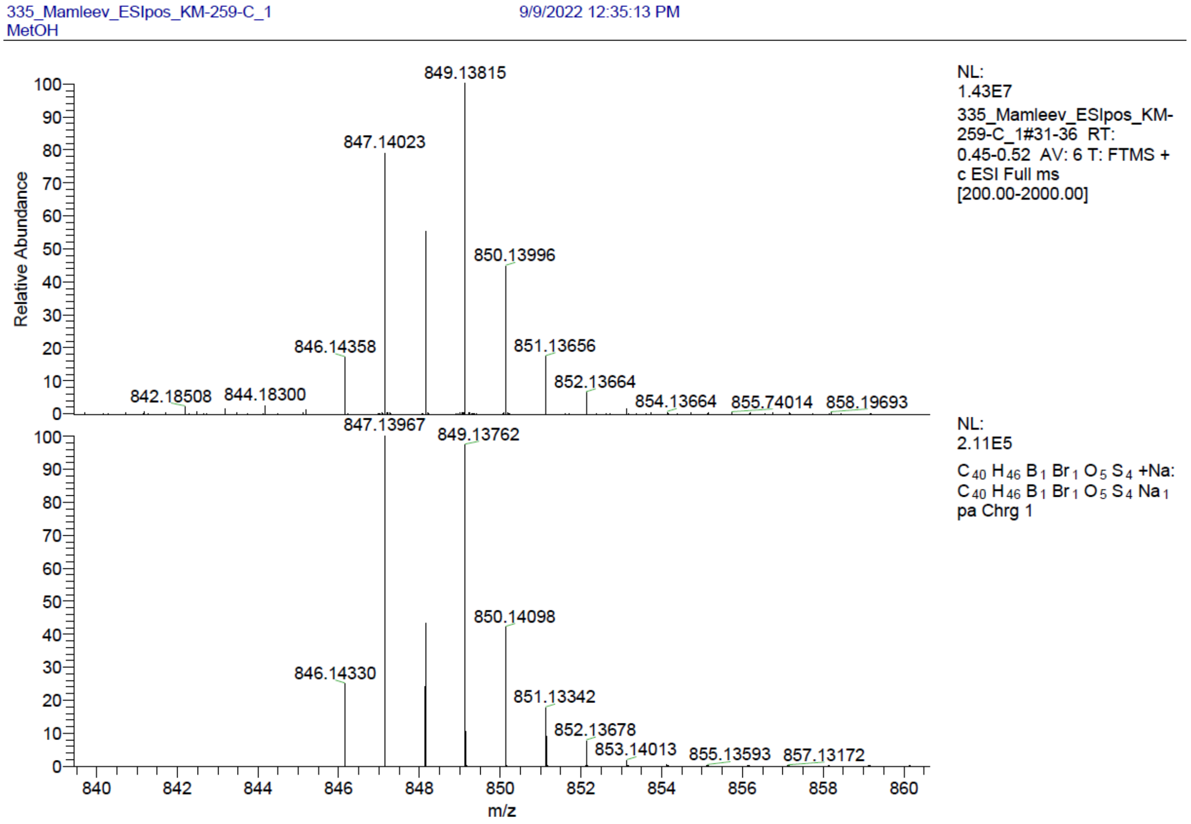

Figure S7: HRMS of compound **5** (ESI$^+$).

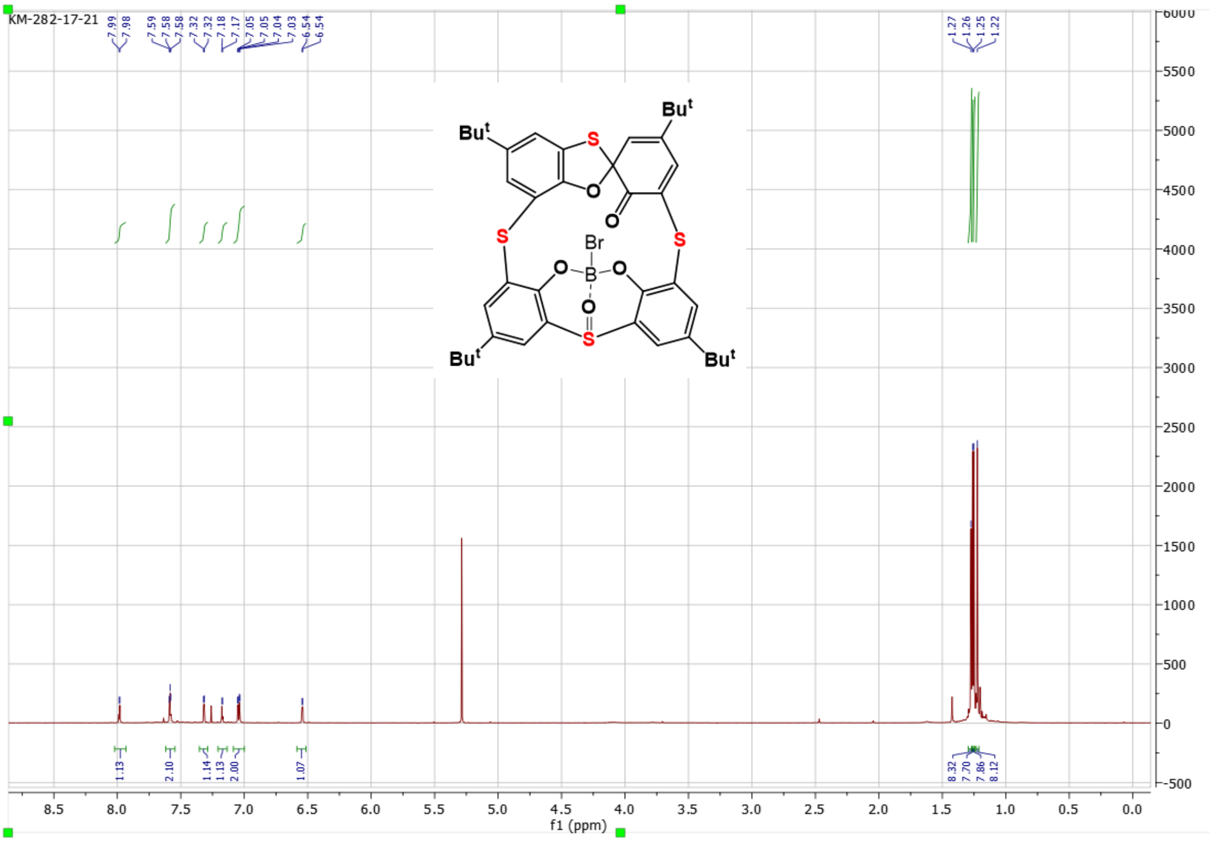

Figure S8: ^{1}H NMR of compound **6** ($CDCl_3$, 400 MHz).

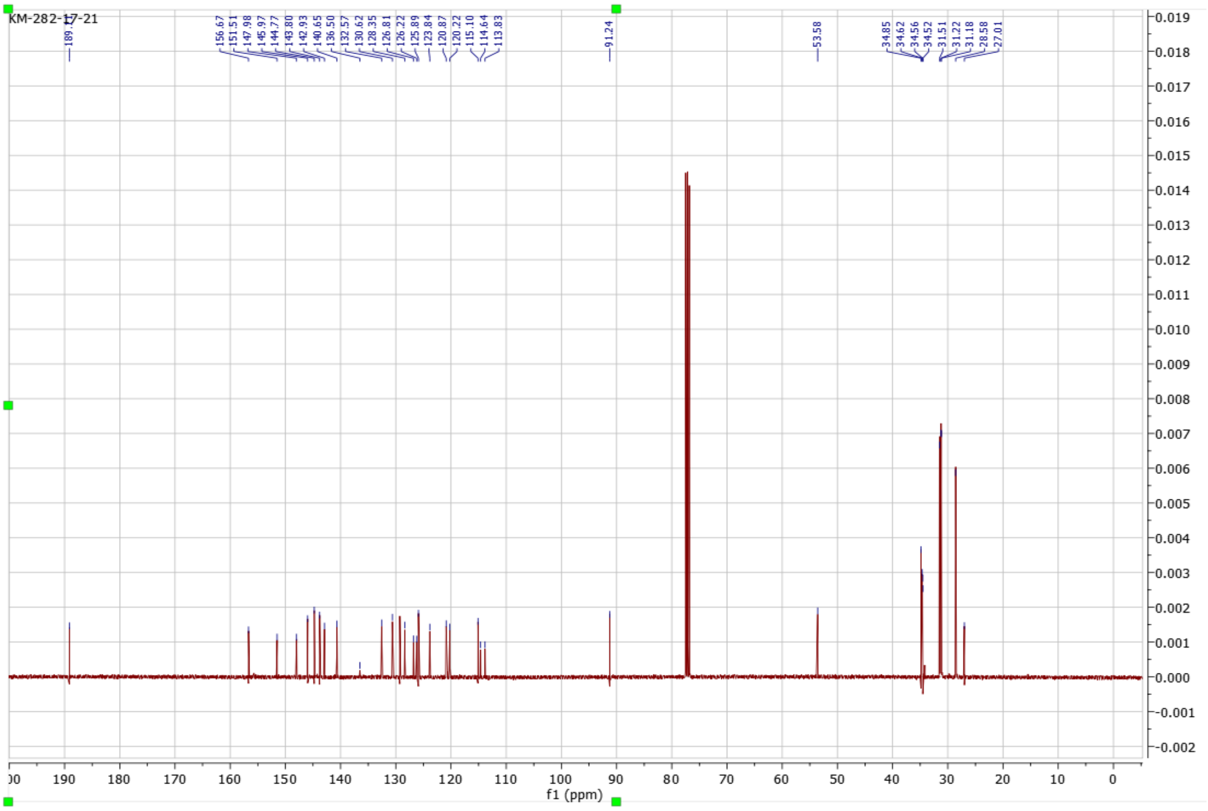

Figure S9: ^{13}C NMR of compound **6** ($CDCl_3$, 100 MHz)

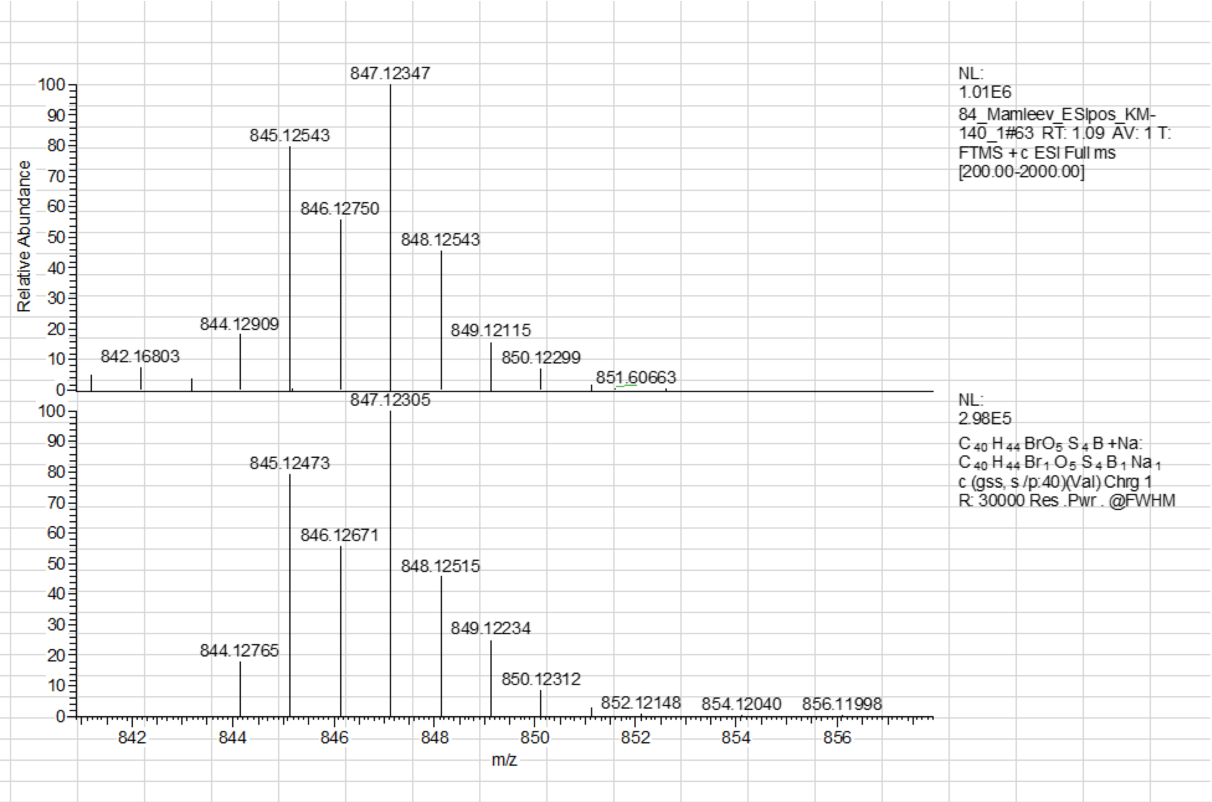

Figure S10: HRMS of compound **6** (ESI+).

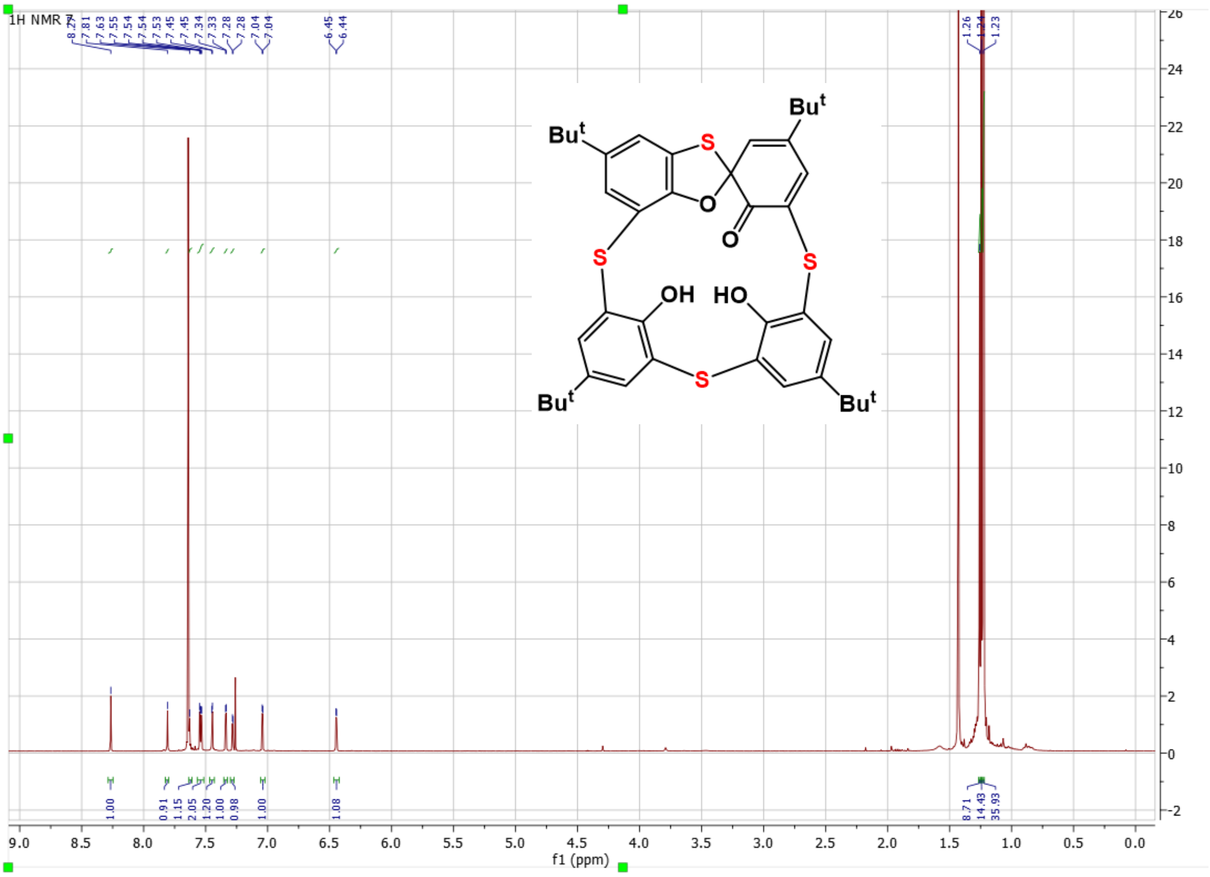

Figure S11: ^{1}H NMR of compound **7** ($CDCl_3$, 400 MHz).

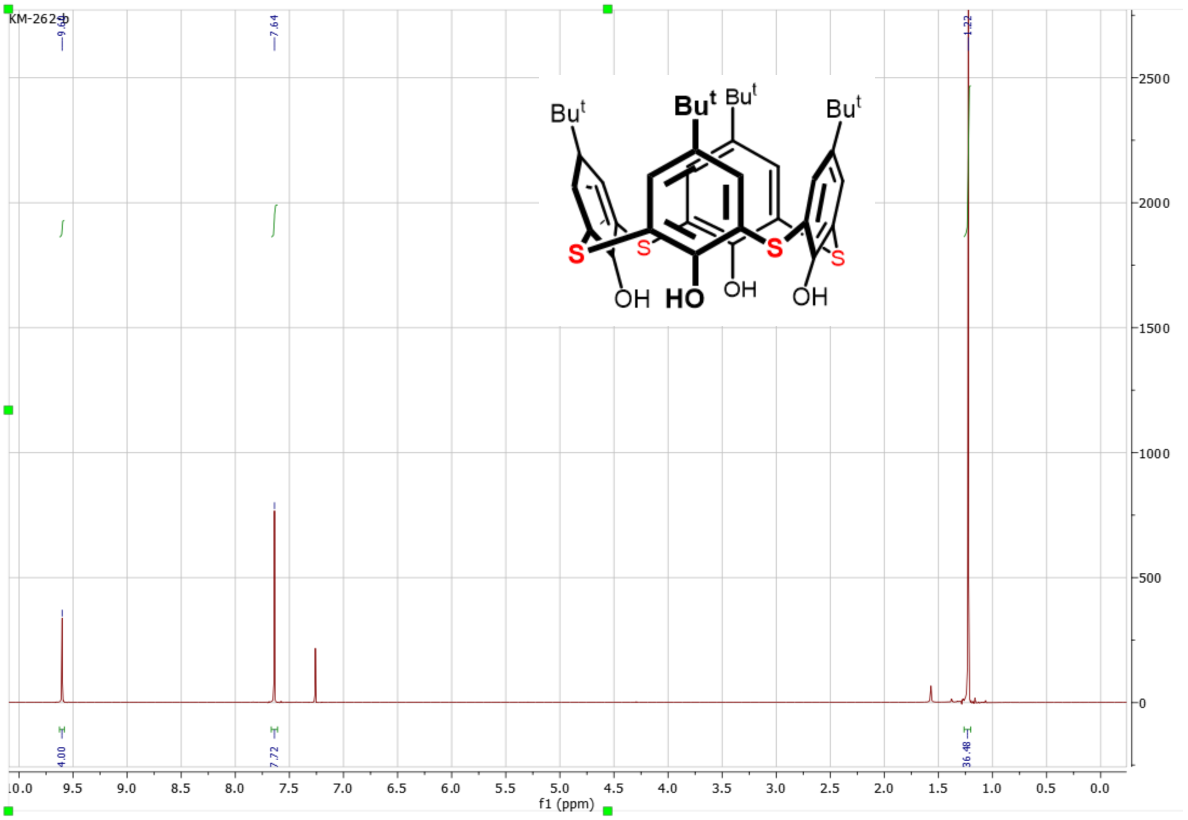

Figure S12: ^{1}H NMR of compound **1** ($CDCl_3$, 400 MHz).

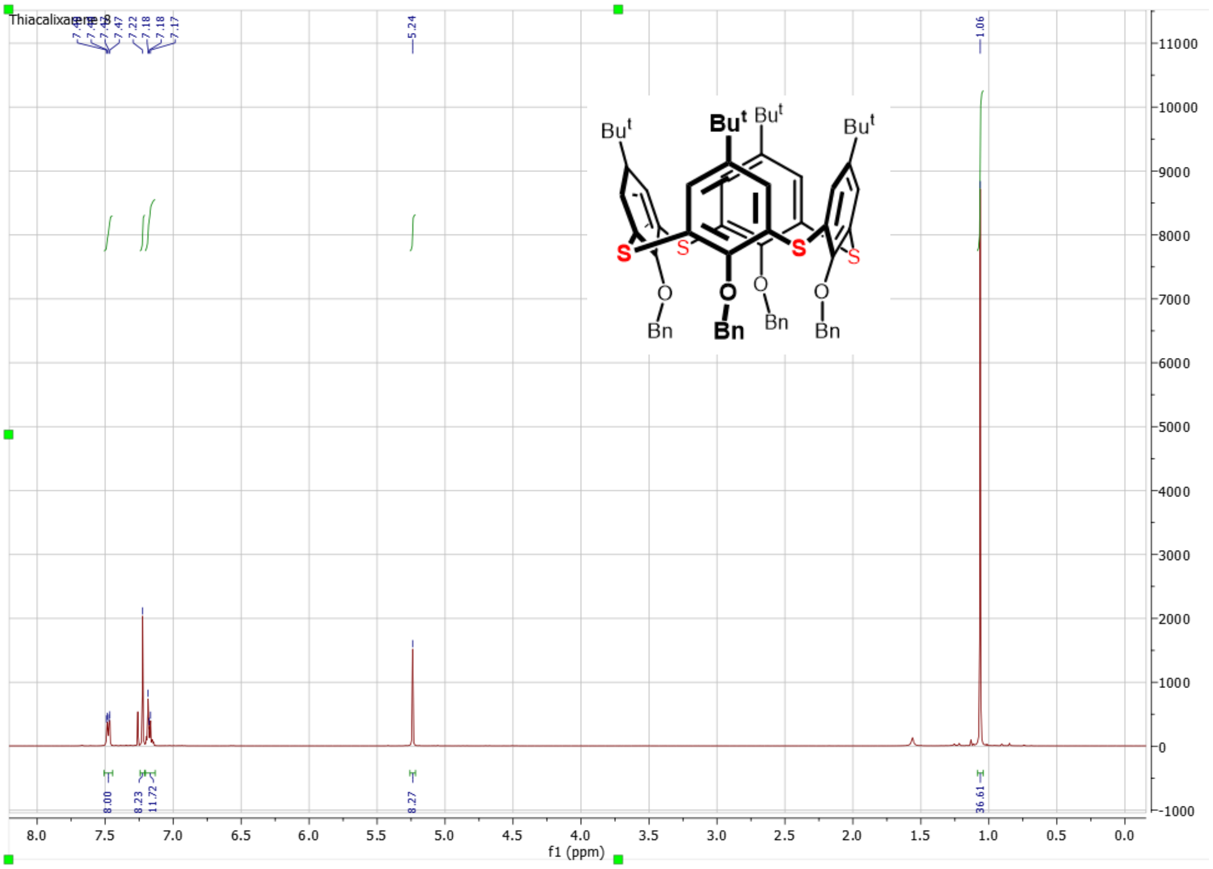

Figure S13: ^{1}H NMR of compound **8** ($CDCl_3$, 400 MHz).

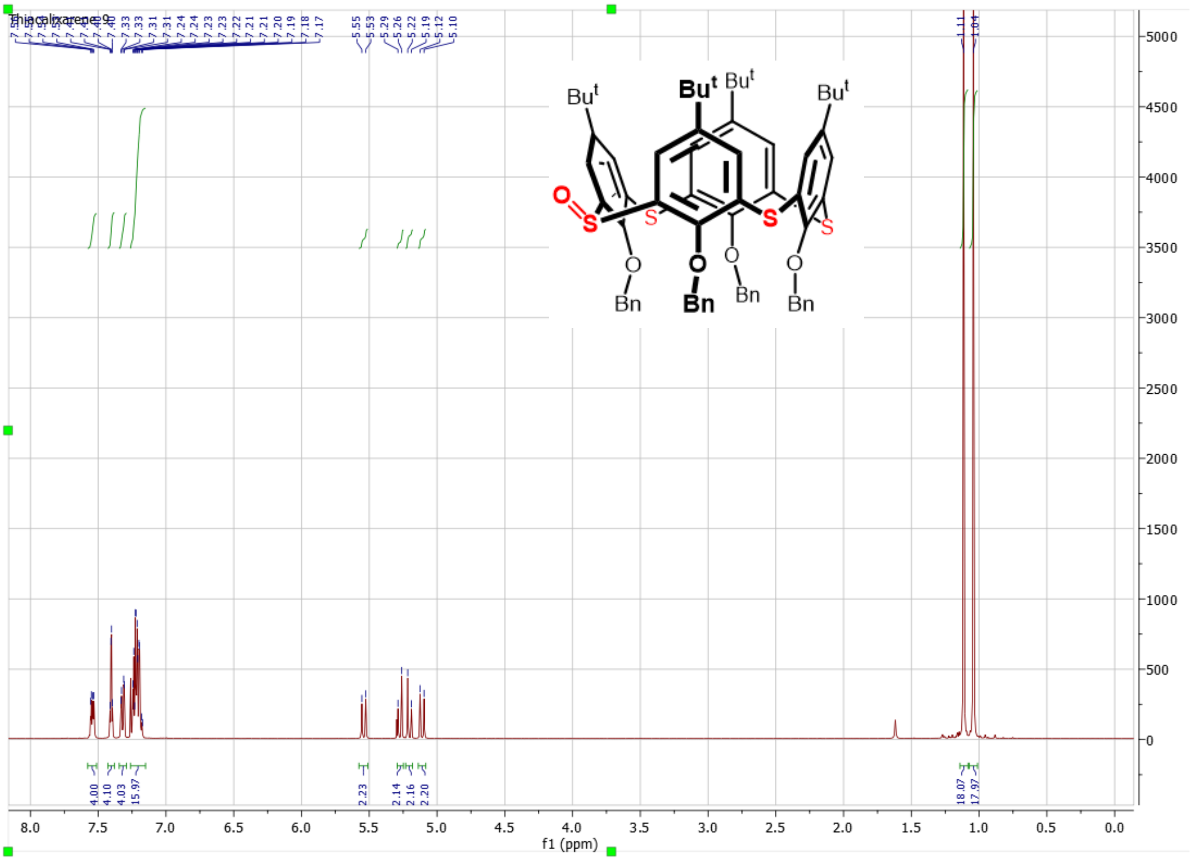

Figure S14: ^{1}H NMR of compound **9** ($CDCl_3$, 400 MHz).

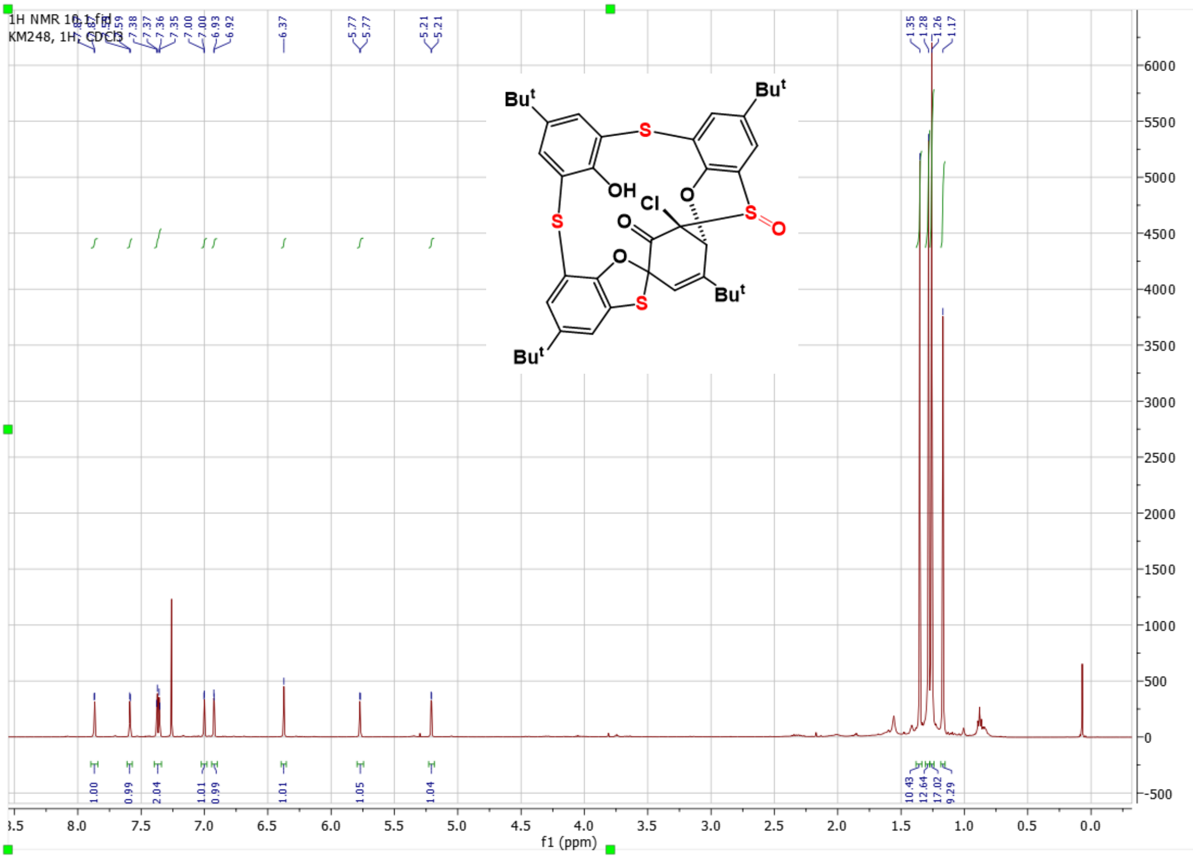

Figure S15: ^{1}H NMR of compound **10** ($CDCl_3$, 400 MHz).

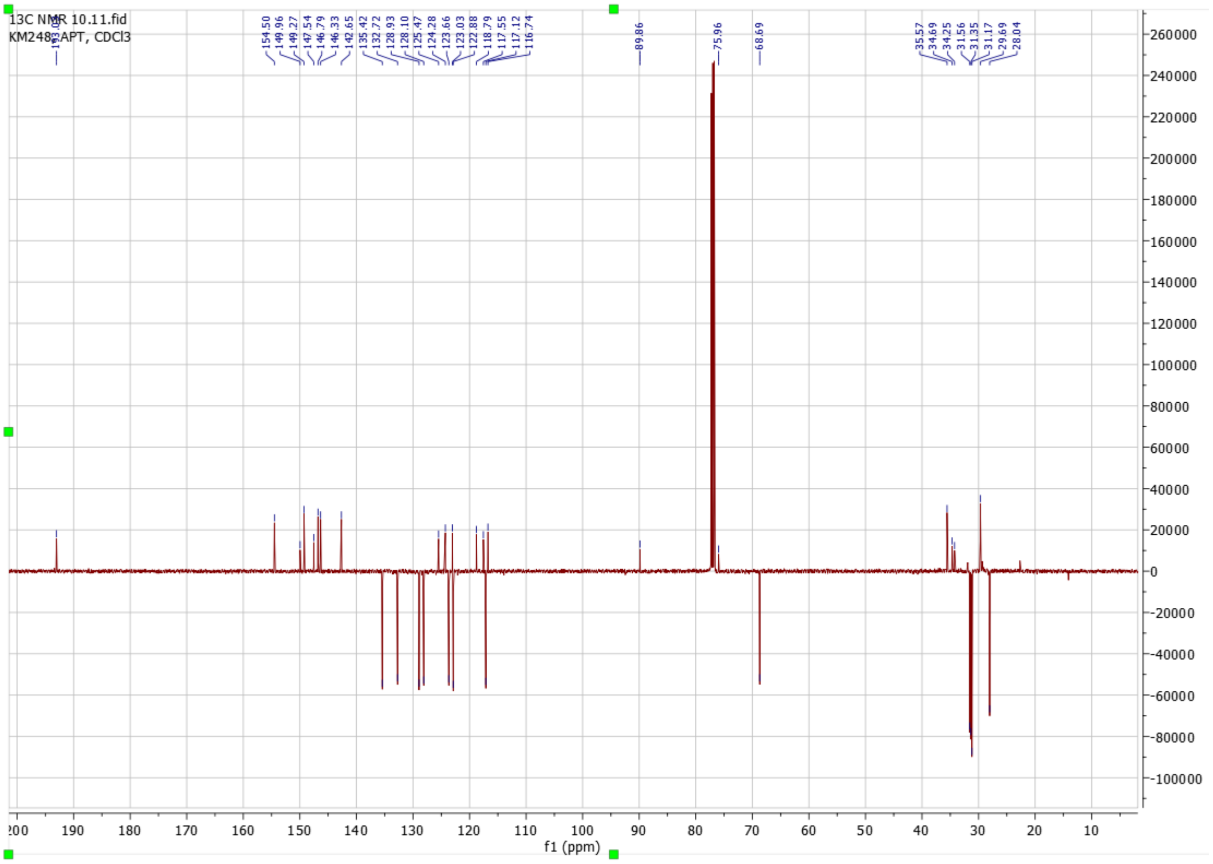

Figure S16: ^{13}C(APT) NMR of compound **10** ($CDCl_3$, 100 MHz)

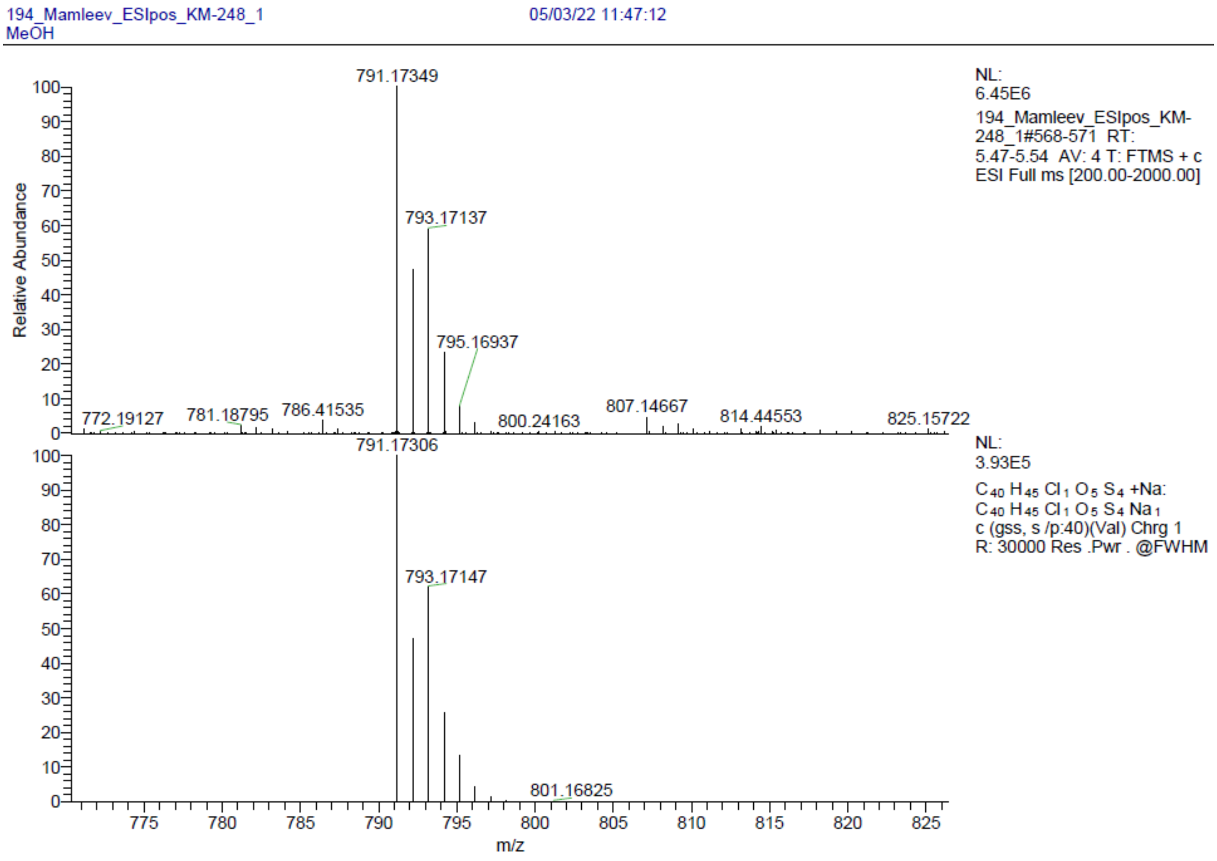

Figure S17: HRMS of compound **10** (ESI$^+$).

2. The Variable Temperature (VT) NMR experiments

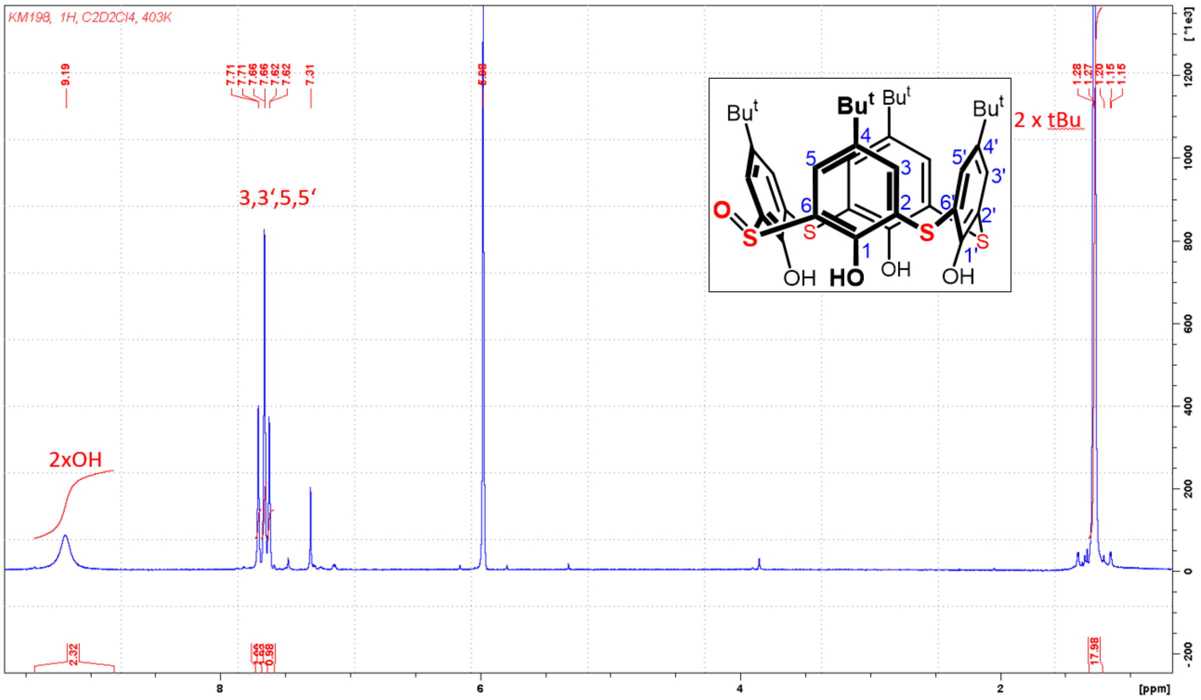

Figure S18: ^{1}H NMR of compound **4** ($C_2D_2Cl_4$, 500 MHz, 403 °K)

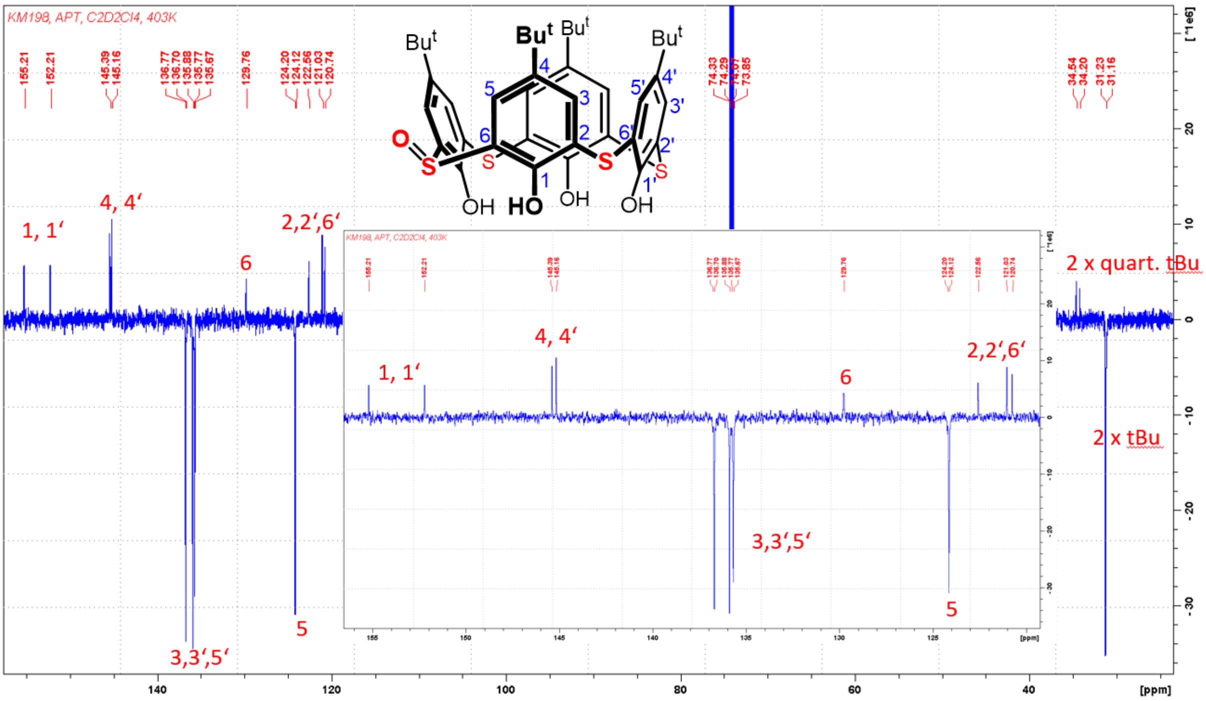

Figure S19: ^{13}C NMR of compound **4** ($C_2D_2Cl_4$, 100 MHz, 403 °K)

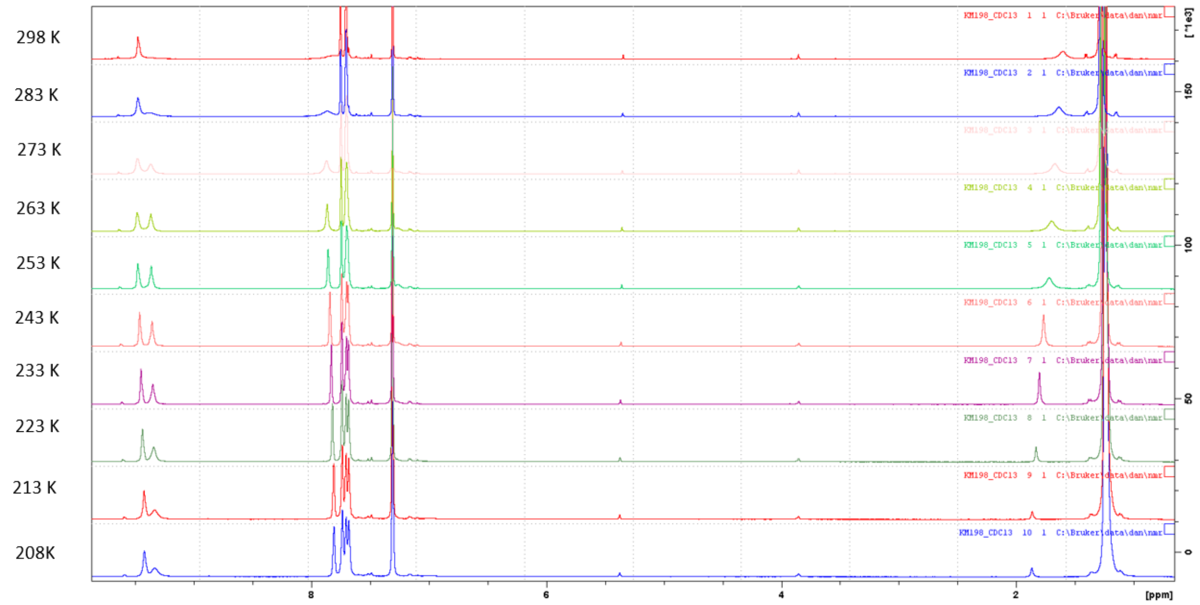

Figure S20: ^{1}H NMR of compound **4** ($CDCl_3$, 500 MHz).

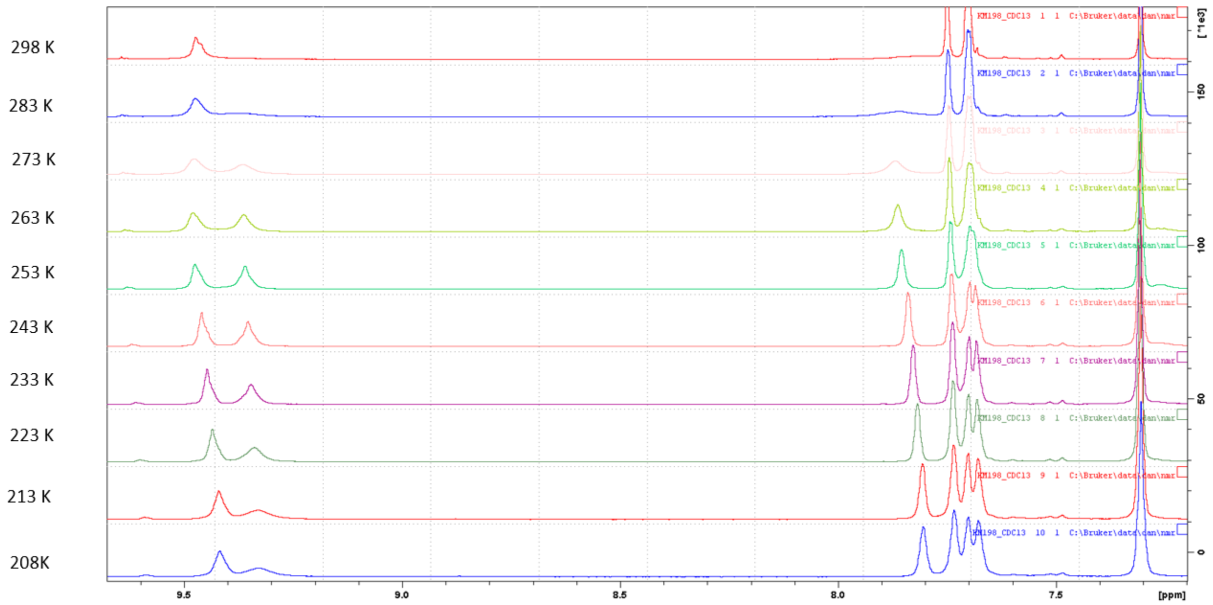

Figure S21: ^{1}H NMR of compound **4** (Aromatic part) ($CDCl_3$, 500 MHz).

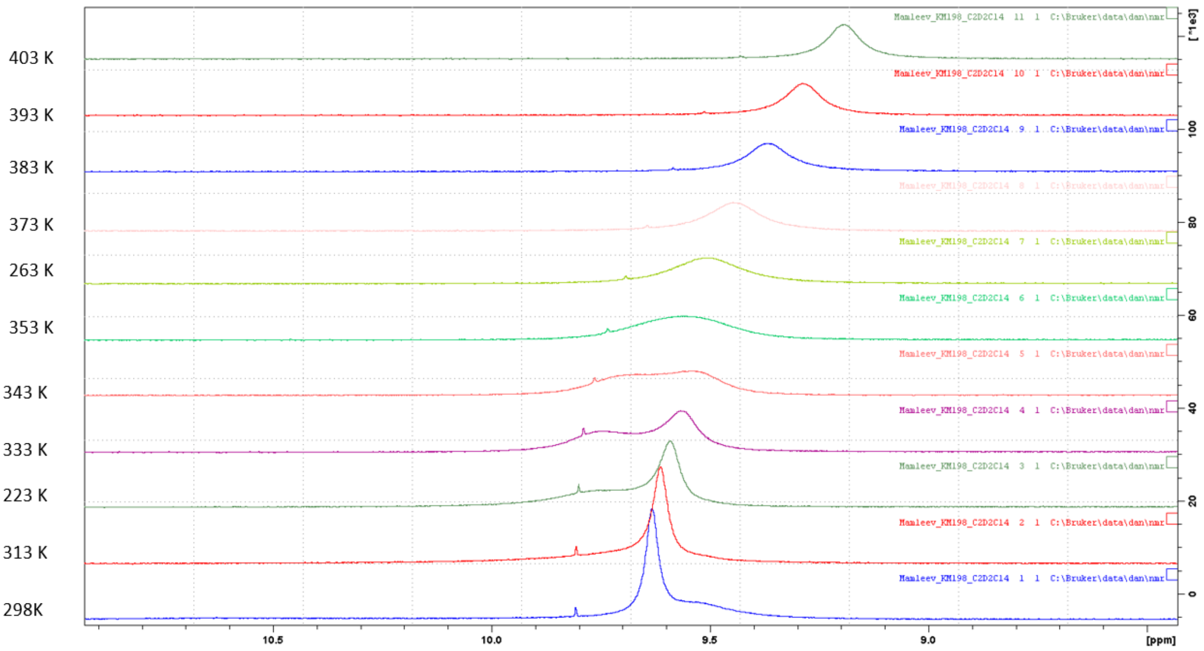

Figure S22: ^{1}H NMR of compound **4** (*OH*- groups part) ($CDCl_3$, 500 MHz).

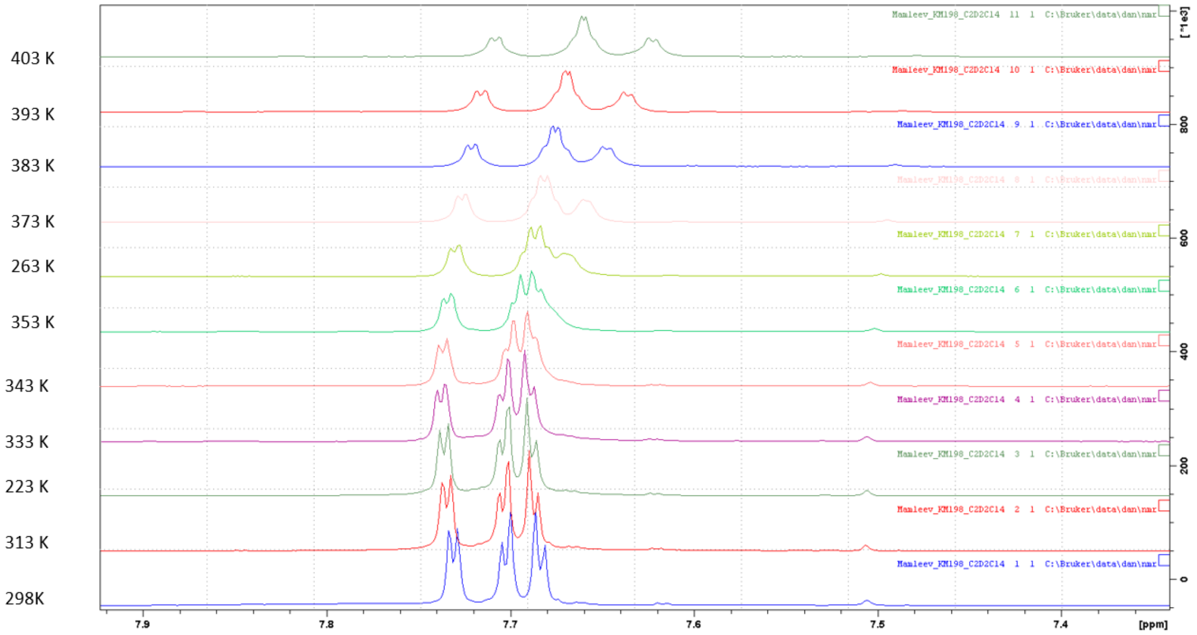

Figure S23: ^{1}H NMR of compound **4** (Aromatic part) ($CDCl_3$, 500 MHz).

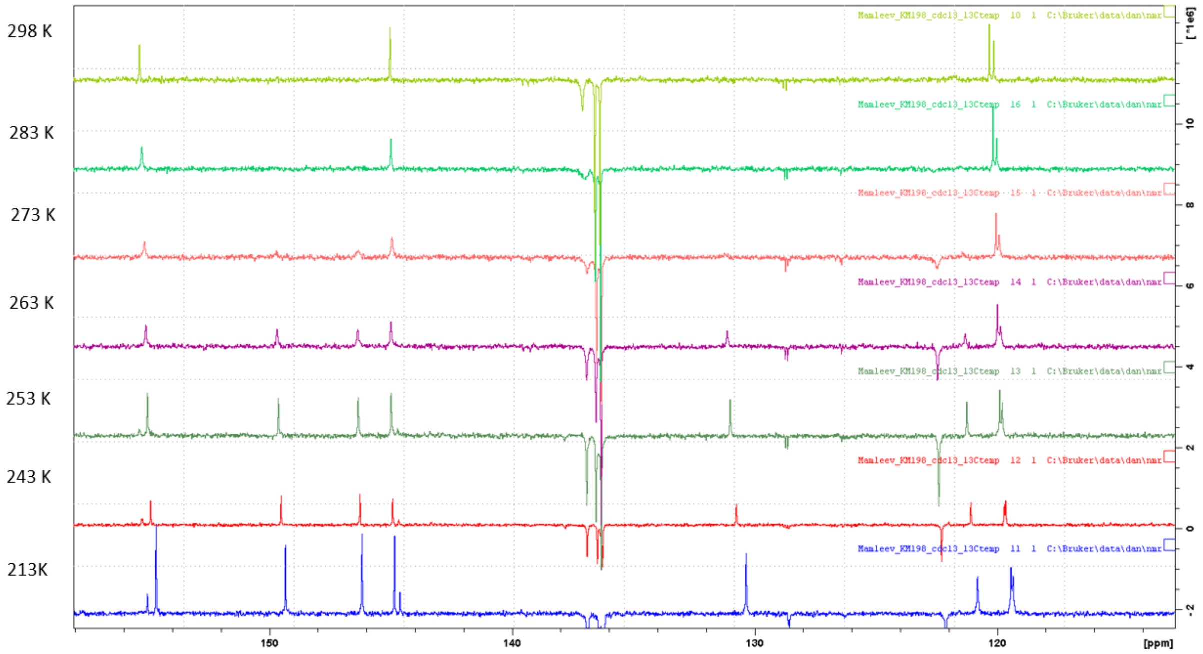

Figure S24: ^{13}C(APT) NMR of compound **4** (Aromatic part) ($CDCl_3$, 100 MHz)

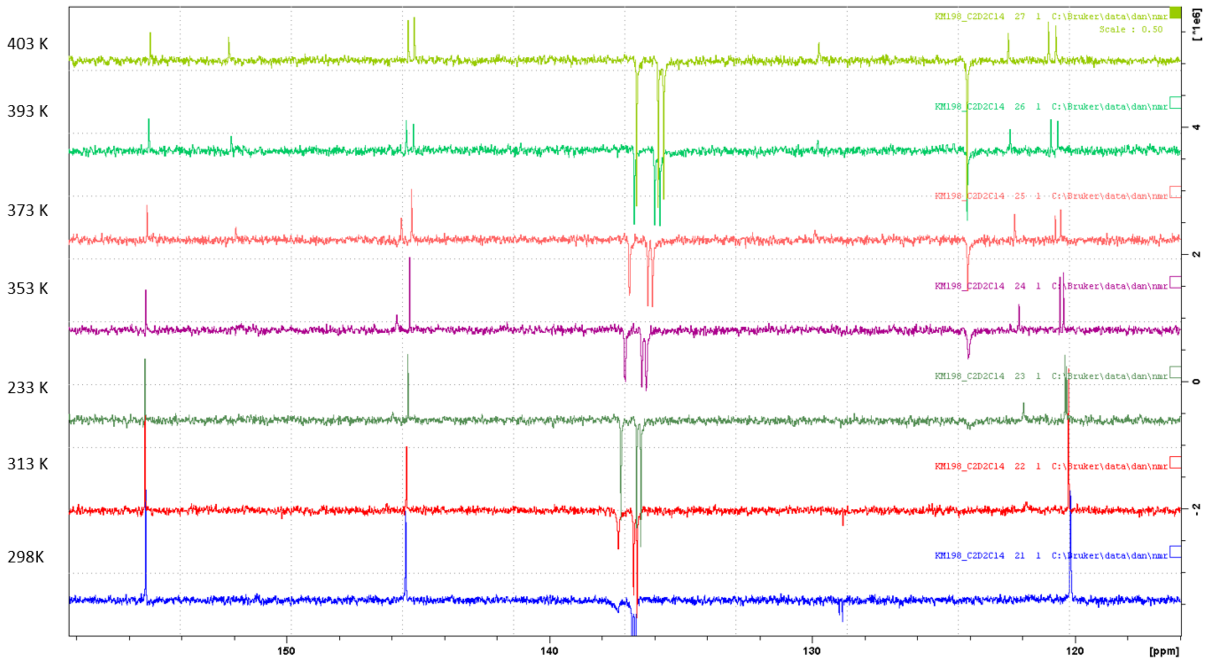

Figure S25: ^{13}C(APT) NMR of compound **4** (Aromatic part) ($CDCl_3$, 100 MHz)

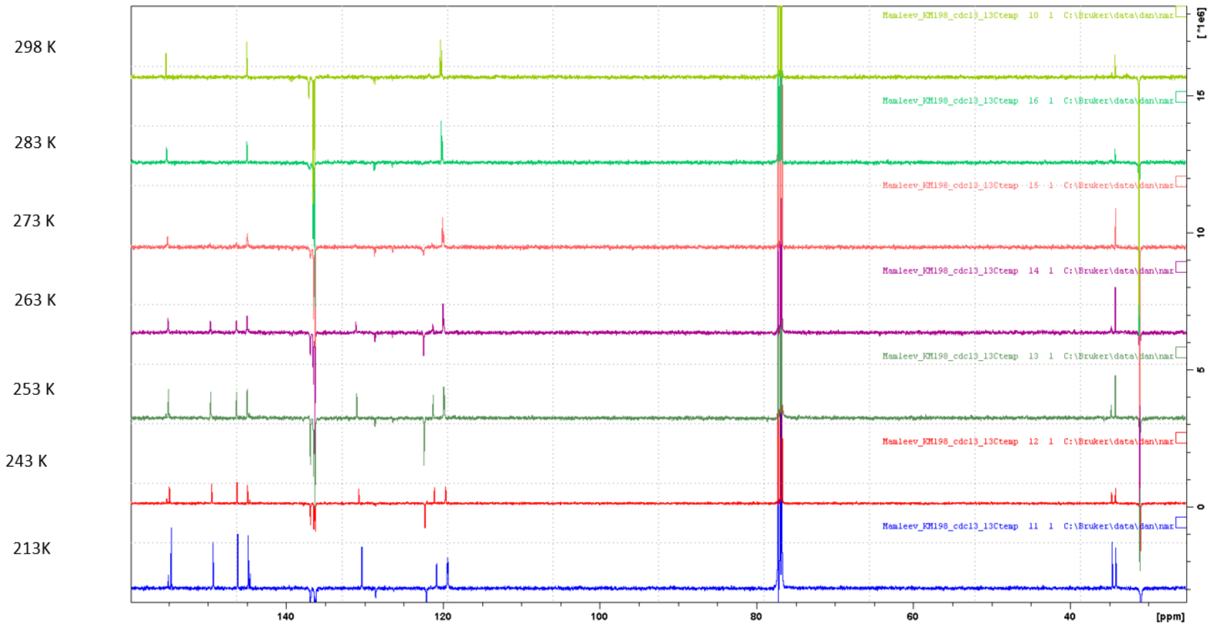

Figure S26: ^{13}C(APT) NMR of compound **4** ($CDCl_3$, 100 MHz)

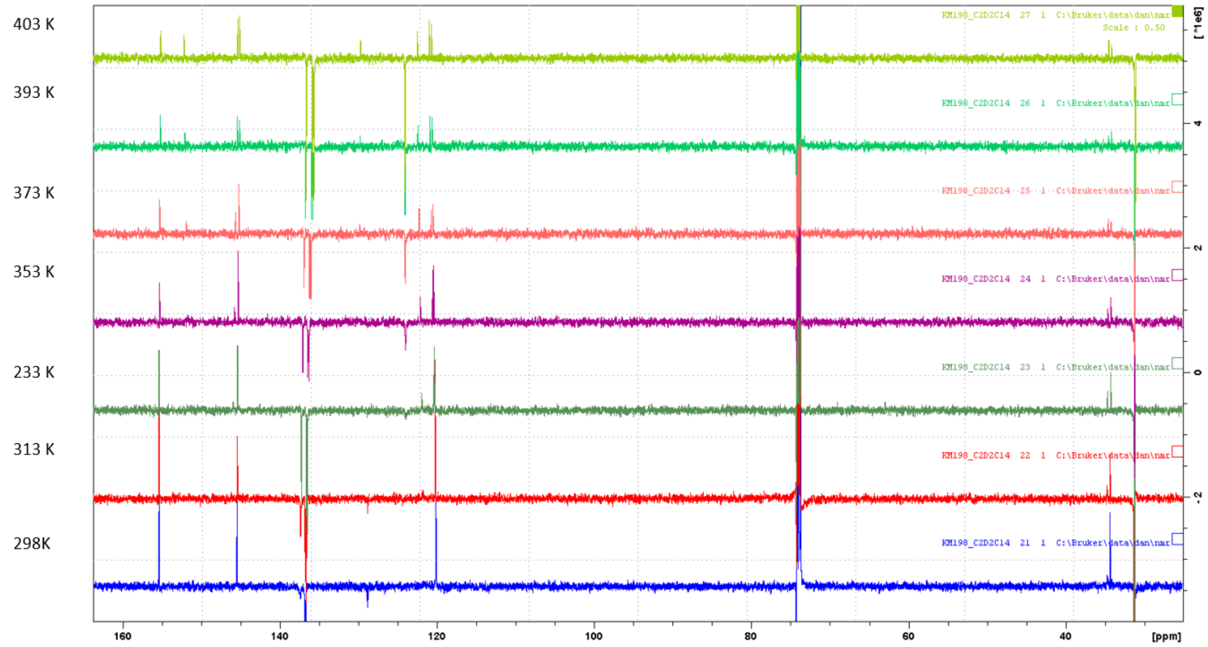

Figure S27: ^{13}C(APT) NMR of compound **4** ($CDCl_3$, 100 MHz)

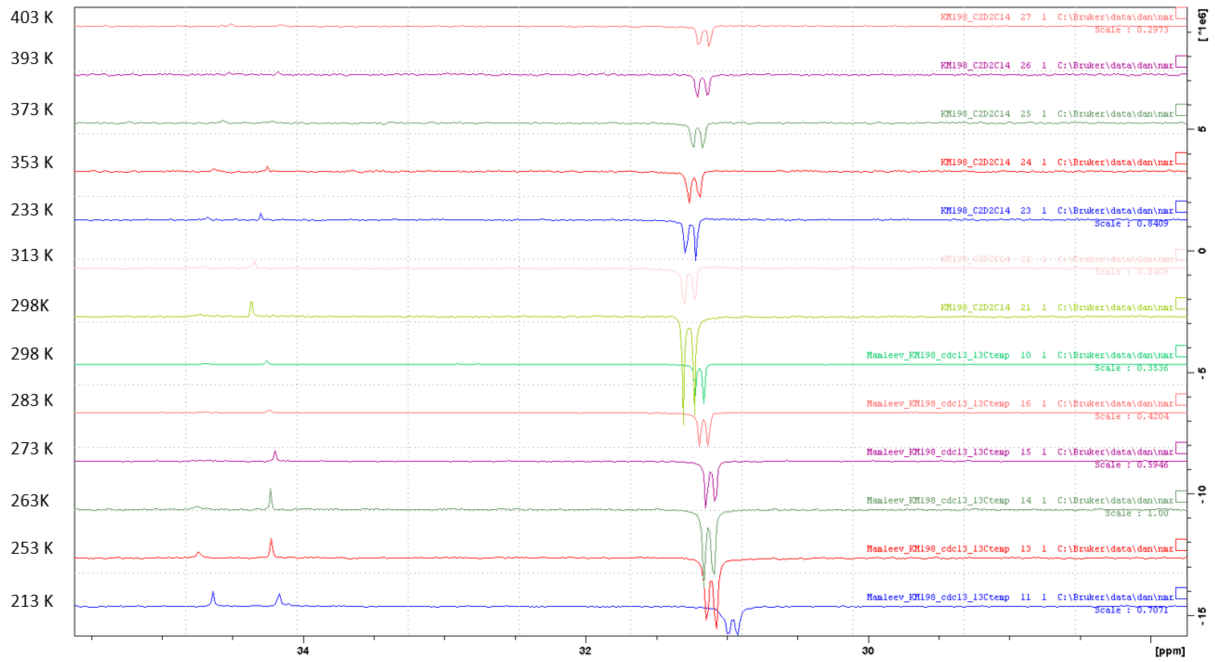

Figure S28: ^{13}C(APT) NMR of compound **4** (*tert*-butyl part) ($CDCl_3$, 100 MHz)

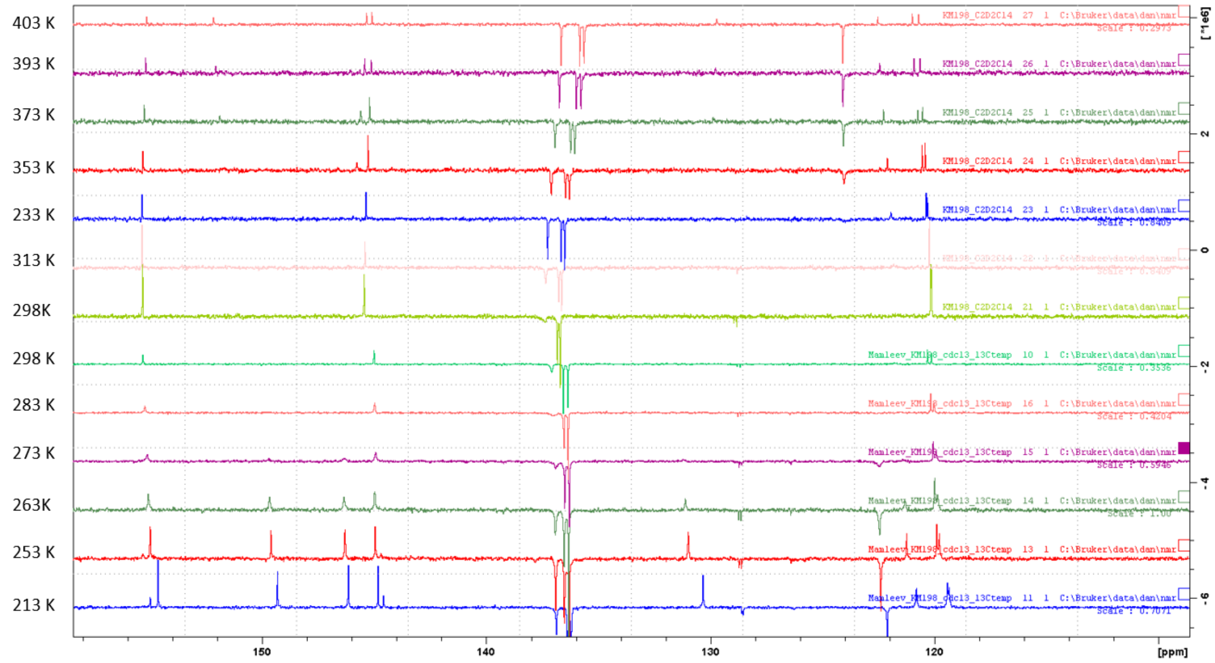

Figure S29: ^{13}C(APT) NMR of compound **4** (Aromatic part) ($CDCl_3$, 100 MHz)

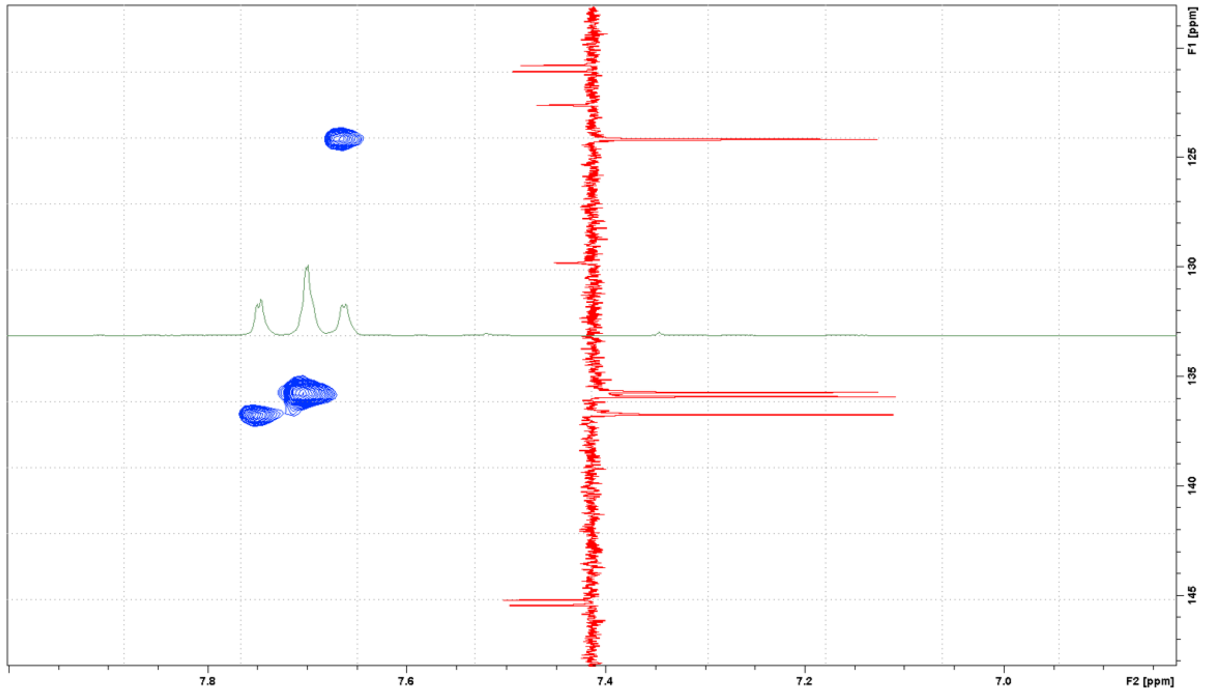

Figure S30: HMQC NMR of compound **4** (Aromatic part, 403 °K) ($CDCl_3$, 100 MHz)

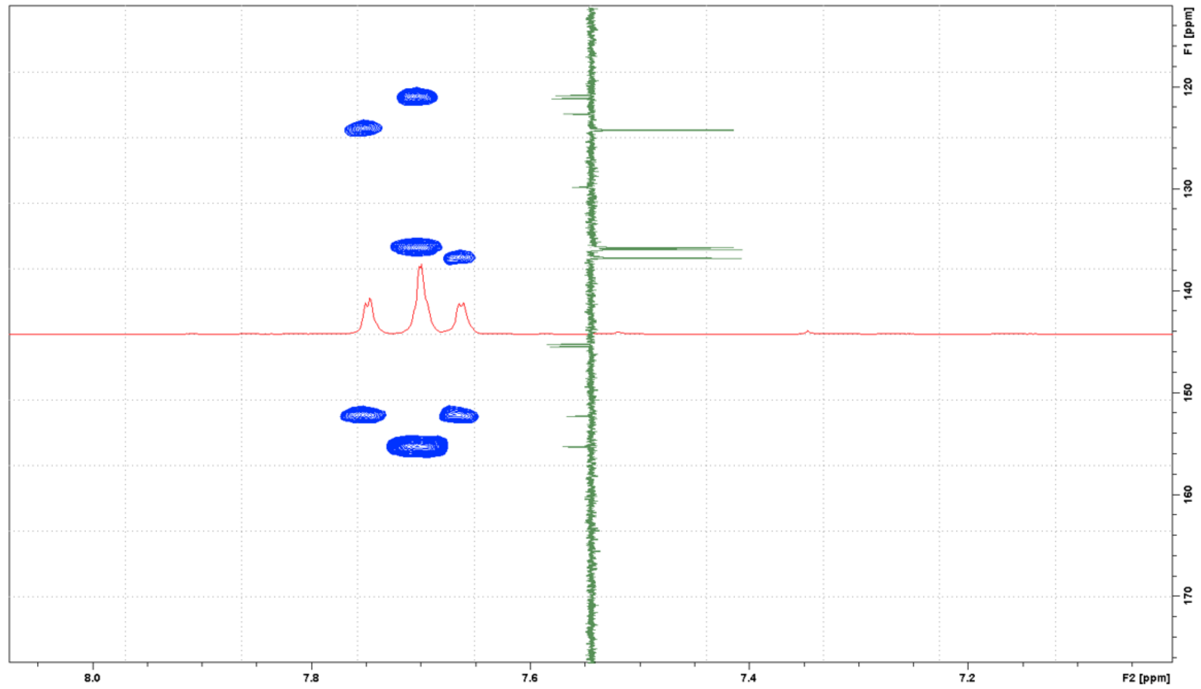

Figure S31: HMBC NMR of compound **4** (Aromatic part 403 °K) ($CDCl_3$, 100 MHz)

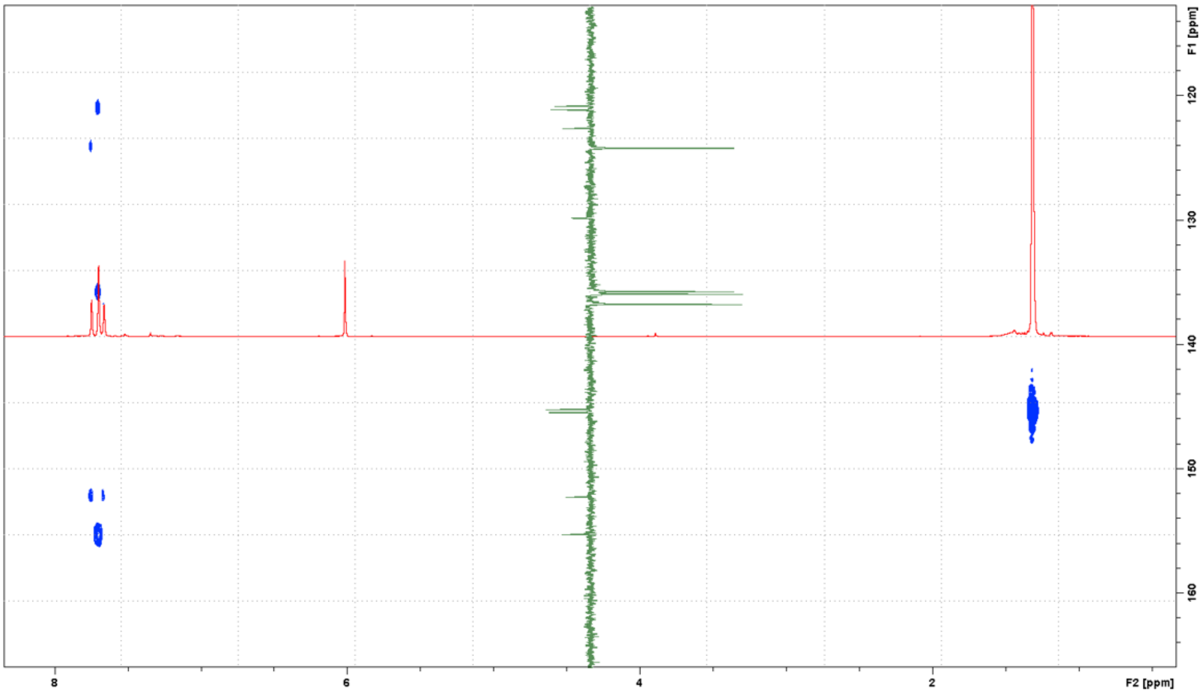

Figure S32: HMBC NMR of compound **4** (Aromatic and *tert*-butyl parts, 403 °K) ($CDCl_3$, 100 MHz)

3. Crystallographic data

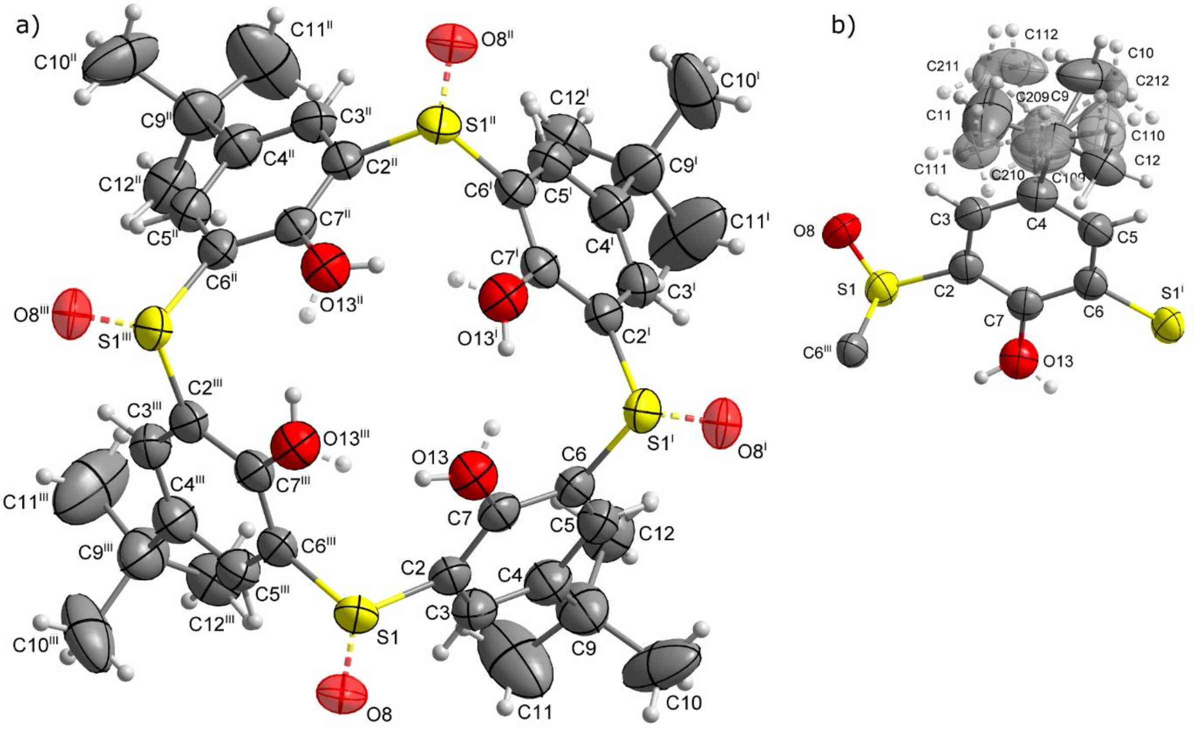

Figure S33: Numbering scheme of crystal structure **4**, with a) full calixarene visible, disordered tert-butyl groups omitted for clarity and b) the asymmetric unit with tert-butyl group disorder visible. Weakly occupied atoms depicted as transparent with dashed bonds. Symmetry codes: (i) 0.5-y, x, z; (ii) 0.5-x, 0.5-y, z; (iii) y, 0.5-x, z.

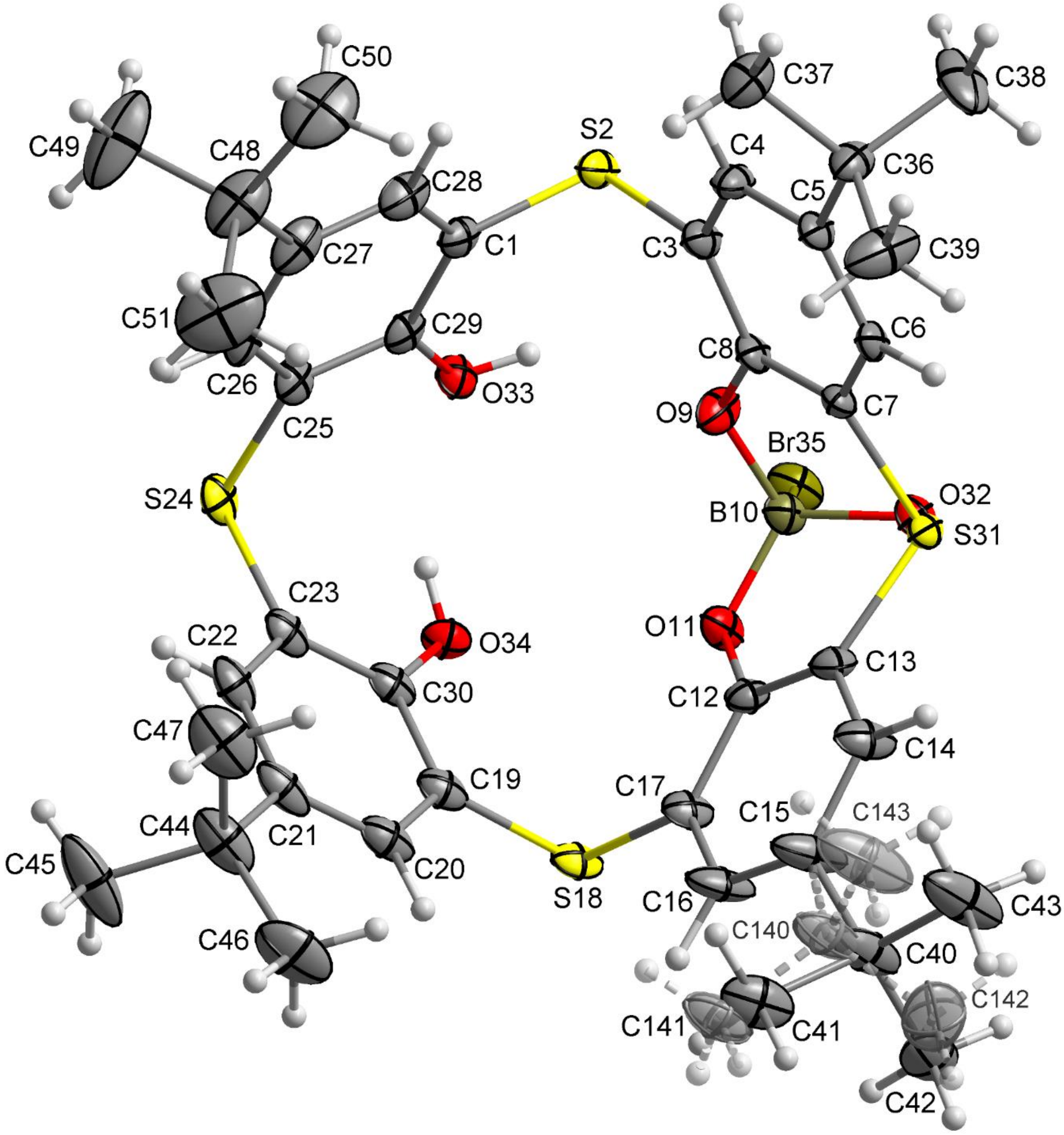

Figure S34: Numbering scheme of crystal structure **5**. Weakly occupied atoms depicted as transparent with dashed bonds.

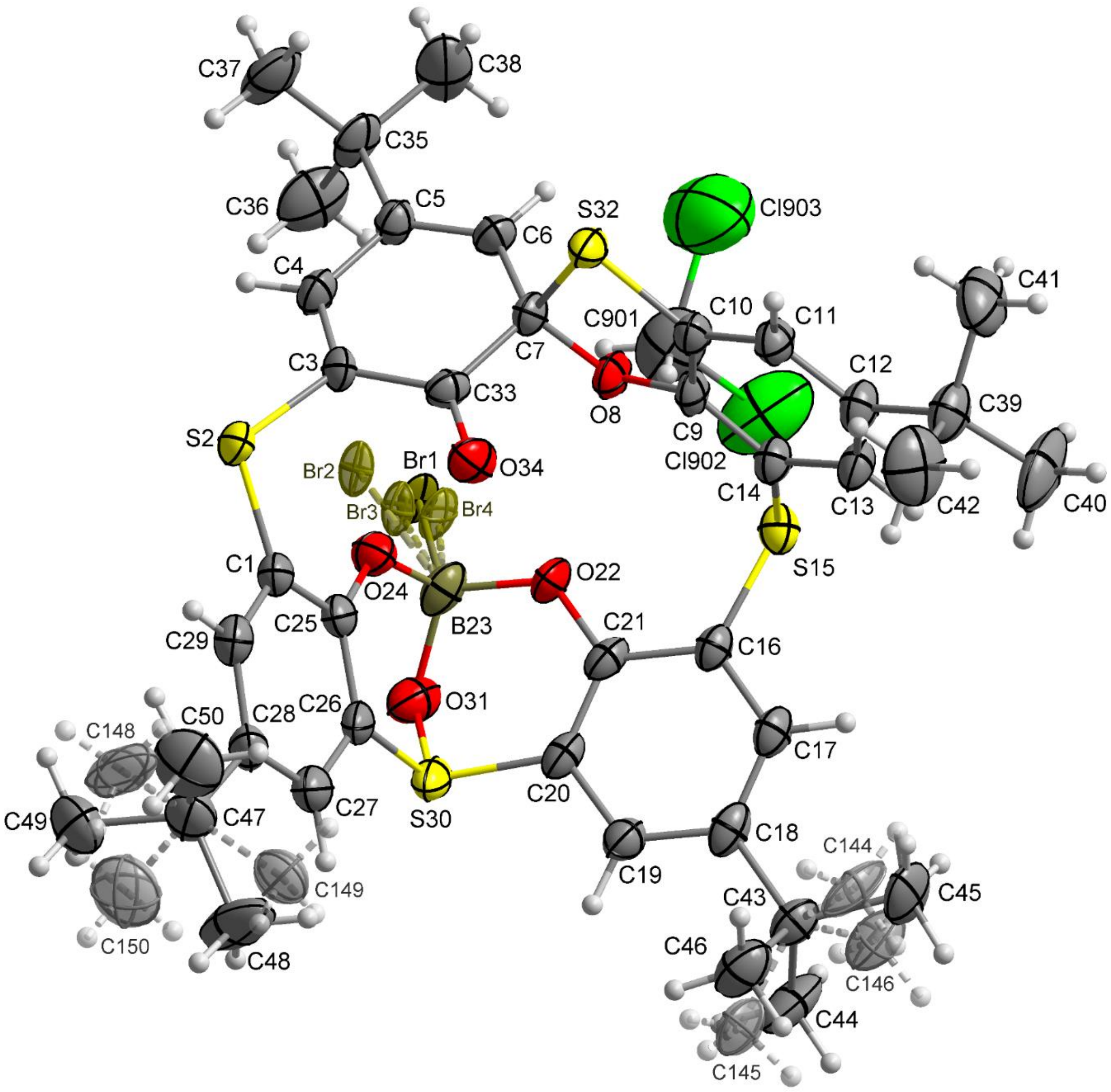

Figure S35: Numbering scheme of crystal structure **6**. Weakly occupied atoms depicted as transparent with dashed bonds.

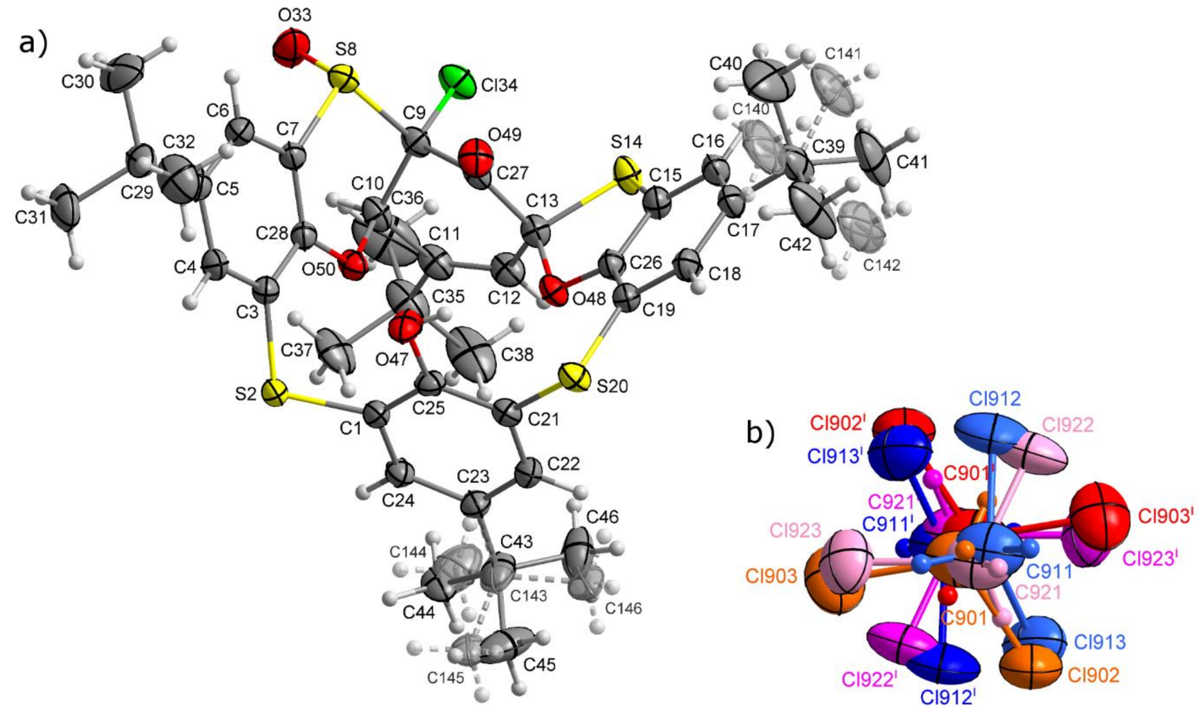

Figure S36: Numbering scheme of crystal structure **10** with a) disordered solvent omitted b) solvent disorder with color-coding. Weakly occupied atoms depicted as transparent with dashed bonds. Symmetry code: (i) -x, 2-y, -z.
